# Video-rate volumetric chemical imaging via mid-infrared photothermal optical diffraction tomography

**DOI:** 10.1038/s41467-026-78699-z

**Published:** 2026-10-30

**Authors:** Masato Fukushima, Keiichiro Toda, Yusei Sugawara, Shotaro Kawano, Takuro Ideguchi

**Affiliations:** 1 Department of Physics, The University of Tokyo, Tokyo, Japan; 2 Institute for Photon Science and Technology, The University of Tokyo, Tokyo, Japan; 3 Department of Advanced Materials Science, The University of Tokyo, Kashiwa, Japan; 4 RIKEN Center for Advanced Photonics, RIKEN, Wako, Japan

**Keywords:** Microscopy, Microscopy

## Abstract

Label-free vibrational microscopy provides chemically specific access to cellular structures, yet quantitative volumetric imaging of intracellular dynamics remains challenging on subsecond timescales. Here, we demonstrate video-rate mid-infrared photothermal optical diffraction tomography (MIP-ODT) by combining high photothermal sensitivity with rapid angular scanning. The system achieves 19.2 volumes per second, nearly 400 times faster than previous MIP tomography implementations, with a signal-to-noise ratio exceeding 70 and no temporal averaging. We track lipid droplets in living cells and quantify anomalous diffusion from three-dimensional trajectories, revealing heterogeneous intracellular transport behaviors obscured in two-dimensional measurements. Rapid mid-infrared wavenumber sweeping further enables broadband multi-wavenumber volumetric chemical imaging across a 300 cm⁻¹ spectral window within 1 s. These results establish MIP-ODT as a platform for high-speed, label-free volumetric chemical imaging of single cells and quantitative investigation of dynamic intracellular organization.

## Abstract (web summary)

Chemical imaging can reveal cellular structures without labels, but capturing their rapid 3D dynamics remains challenging. Here, the authors develop mid-infrared photothermal microscopy for video-rate 3D chemical imaging, revealing diverse lipid droplet movements in living cells.

## Introduction

In life science, volumetric imaging provides three-dimensional (3D) access to subcellular architecture and dynamic processes, supporting diverse biomedical studies spanning from organelle transport to mapping of tissue microenvironments^1,2^. Among existing live-cell imaging techniques, 3D fluorescence microscopy is widely regarded as the gold standard due to its high image contrast and molecular specificity^3^. However, fluorescence-based approaches rely on exogenous labels, which can perturb native biological states, complicate sample preparation, and restrict the number of molecular species that can be simultaneously interrogated. Label-free vibrational microscopy offers a complementary route by directly extracting chemically specific information from endogenous molecular vibrations. Coherent Raman microscopy, in particular, has been widely adopted because it enables rapid two-dimensional (2D) imaging with high spatial resolution, making it well suited for live-cell chemical imaging^4^. Extending coherent Raman microscopy to volumetric imaging has been achieved by incorporating axial scanning, enabling 3D chemical visualization in diverse contexts such as lipid droplet organization^5^, myelin-rich neural tissue^6^, and metabolic heterogeneity in multicellular assemblies^7^. Despite these advances, volumetric Raman imaging remains fundamentally constrained in throughput. The requirement for sufficient signal-to-noise ratio (SNR) typically necessitates point-scanning acquisition, imposing a fundamental trade-off between imaging speed and sampled volume. Consequently, even the fastest reported implementations are limited to whole-cell volumetric imaging rates of approximately one volume per second (vps), which is inadequate for resolving many intracellular dynamics that occur on sub-second timescales^8,9^.

In parallel, recent advances have expanded the technical landscape of mid-infrared (MIR) chemical imaging^10,11^. Among these approaches, mid-infrared photothermal (MIP) microscopy has recently emerged as a powerful approach for high-speed chemical imaging that overcomes the diffraction limit of MIR light^12–22^. In MIP microscopy, MIR absorption induces local refractive index (RI) changes that are detected by a visible probe beam, enabling bond-selective chemical imaging with visible diffraction-limited resolution. Because MIR absorption is a linear process, MIP microscopy can be implemented in both point-scanning and wide-field configurations, and recent advances have demonstrated video-rate 2D MIP imaging in both modalities^20,21^. In wide-field MIP, photothermal contrast is captured over the entire field of view within a single camera exposure. This spatial parallelism makes wide-field MIP, in principle, well suited for further speed scaling and suggests a promising pathway toward video-rate volumetric imaging. 3D information can be obtained by integrating wide-field MIP with diffraction tomography techniques, such as optical diffraction tomography (ODT)^17^ or intensity diffraction tomography (IDT)^19^, which reconstruct 3D RI distributions from amplitude-and phase- or intensity-only measurements acquired under multiple visible illumination angles. A key advantage of diffraction tomography is its flexible trade-off between imaging throughput and reconstruction fidelity. Imaging speed can be increased by reducing angular sampling density without decreasing the sampled volume, making this framework particularly attractive for high-speed volumetric chemical imaging. Despite this potential, previously reported implementations of wide-field MIP tomography have achieved volumetric imaging rates below 0.05 vps^17,19^. This limitation arises because existing approaches either suffer from insufficient single-frame SNR at each illumination angle, necessitating frame averaging, or are limited by the switching speed of the visible illumination angles. As a result, a substantial gap remains between current MIP-based tomographic methods and video-rate volumetric chemical imaging.

In this work, we establish a fundamentally distinct performance regime in MIP tomography by jointly advancing per-frame MIP detection sensitivity and angular switching speed, thereby transcending the conventional speed-sensitivity trade-off and enabling video-rate volumetric chemical imaging. We implement this strategy within an MIP-ODT framework. High MIP sensitivity is achieved by employing a high-power nanosecond-pulsed MIR source generated by an optical parametric oscillator (OPO), which provides sufficient SNR within a single camera exposure at each illumination angle. Rapid angular scanning is realized through fast wavefront modulation using a high-speed spatial light modulator (SLM). Using eleven illumination angles with a numerical aperture (NA) of 0.85, our system achieves a volumetric imaging rate of 19.2 vps, enabling video-rate 3D tracking of lipid droplets in living cells and quantitative analysis of their anomalous diffusion. Furthermore, integration of a fast MIR wavenumber-swept module enables 3D broadband multi-wavenumber spectral imaging, allowing the acquisition of 20 spectral points spanning a 300 cm^−1^ window within approximately one second. Using this capability, we resolved distinct vibrational spectral signatures associated with subcellular structures, including nucleoli and lipid droplets. Together, these advances establish a platform for high-speed, label-free volumetric chemical imaging at the single-cell level and open new opportunities for visualizing dynamic intracellular molecular processes, including drug accumulation, lipid droplet formation and fusion, and the emergence of intracellular aggregates.

## Results

### Video-rate MIP-ODT system

**Fig. 1:**
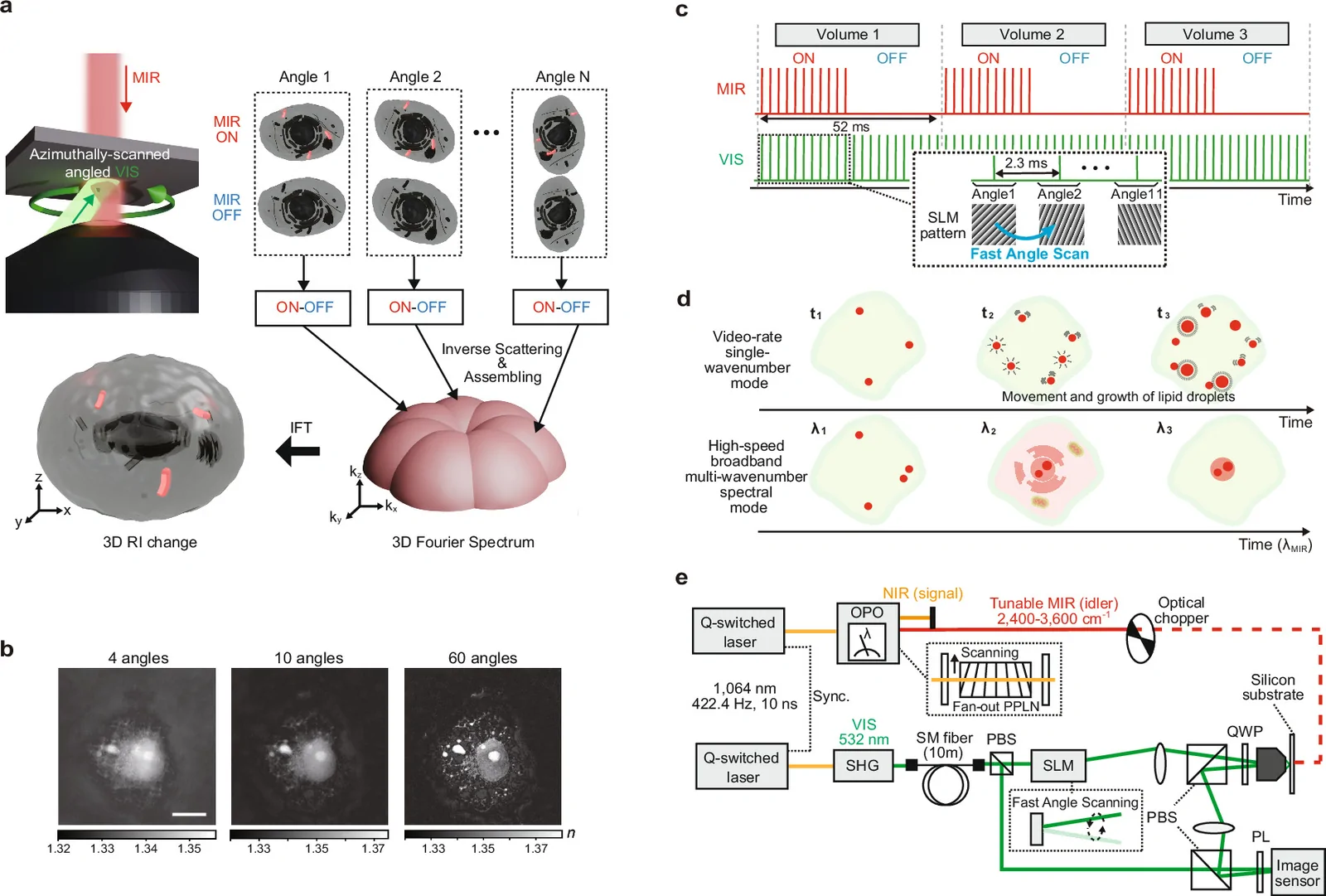
Video-rate MIP-ODT system. **a** A schematic of the MIP-ODT principle. IFT, Inverse Fourier transformation. **b** Reconstructed refractive index (RI) images obtained using different numbers of illumination angles. Scale bar, 10 μm. **c** Timing diagram of video-rate volumetric image acquisition. **d** Operating modes of the system: video-rate single-wavenumber mode and high-speed broadband multi-wavenumber spectral mode. **e** The optical layout of the video-rate MIP-ODT system. The MIR beam is modulated on and off using an optical chopper. MIR mid-infrared; MIP mid-infrared photothermal; VIS visible; NIR near-infrared; SM single-mode; SHG second-harmonic generation; OPO optical parametric oscillator; SLM spatial light modulator; PBS polarizing beam splitter; QWP quarter-wave plate; PL polarizer; PPLN periodically poled lithium niobate; ODT optical diffraction tomography.

We first outline the principle of MIP-ODT. In this method, wide-field MIR irradiation induces localized heating across the field of view through MIR absorption, generating transient RI changes via the thermo-optic effect. These induced RI changes are quantitatively measured by ODT, where 2D optical wavefronts are interferometrically captured under multiple visible illumination angles with and without MIR excitation (Fig. 1a). To reconstruct the 3D distribution of the MIP-induced RI change from the measured wavefront images, the wavefront modulation is extracted for each illumination angle from the difference between MIR-ON and MIR-OFF measurements. Under the weak-scattering approximation, this differential wavefront is mapped to the corresponding spatial-frequency components of the RI change in the sample’s 3D Fourier space. By assembling the spatial-frequency information across all illumination angles, we reconstruct the 3D distribution of MIP-induced RI changes (see “Methods”, Procedure of RI reconstruction).

We then designed an MIP-ODT system for video-rate volumetric imaging. To determine an appropriate balance between imaging speed and reconstruction fidelity, we evaluated reconstructed RI images obtained with different numbers of visible illumination angles (Fig. 1b, see “Methods”, ODT measurement with a variable number of illuminations). Based on this analysis, the number of illumination angles was set to eleven to maximize volumetric acquisition speed while maintaining sufficient image quality to capture global cellular morphology. Figure 1c provides a timing diagram of the video-rate MIP-ODT system. High-speed angular scanning of visible illumination is achieved through fast wavefront control using a high-speed SLM operating at 422.4 Hz, corresponding to an angular update period of 2.3 ms. Under these conditions, the volumetric MIP acquisition rate is given by ${v}_{{{{\rm{ps}}}}}=422.4/(2\cdot 11)$ = 19.2 vps, which corresponds to an effective volumetric acquisition time of 52 ms. Here, the factor of two accounts for the alternating acquisition of MIR-ON and MIR-OFF frames. The system supports two distinct operational modes (Fig. 1d). In the video-rate single-wavenumber mode, volumetric imaging is performed at the full video-rate acquisition speed. In the high-speed broadband multi-wavenumber spectral mode, rapid MIR wavenumber sweeping enables the acquisition of 19.2 spectral points per second. Given that the intrinsic spectral linewidth of the MIR excitation is approximately 10 cm^−^^1^^21^, this configuration enables scanning of a spectral window of several hundred cm^−^^1^ within one second.

Figure 1e shows the optical layout of the video-rate MIP-ODT system. The MIR and visible pulses are derived from two electronically synchronized Q-switched Nd:YAG lasers (wavelength 1064 nm; pulse width, 10 ns; repetition rate, 422.4 Hz; NL202, Ekspla). The visible probe beam is generated via second-harmonic generation (SHG) using a 15-mm-long LBO crystal. The MIR pump beam is provided by the idler output of an OPO incorporating a fan-out periodically poled lithium niobate (PPLN) crystal as the nonlinear medium (HC Photonics Corporation). The MIR output wavenumber is continuously tunable over the range 2400–3600 cm^−1^ by laterally translating the fan-out PPLN crystal using a motorized translation stage (LNR25ZFS/M, Thorlabs), which selectively accesses different poling periods along the crystal. The OPO delivers pulse energies of ~3 μJ at the sample plane, more than one order of magnitude higher than those of quantum cascade laser (QCL) sources used in previous MIP tomography implementations^17,19^. This substantially enhances the single-frame MIP detection sensitivity. The short 10-ns MIR pulses effectively suppress spatial blurring caused by thermal diffusion, limiting heat-induced resolution degradation to ~10 nm^22^. In addition, the sub-kHz repetition rate ensures sufficient thermal relaxation between successive excitation pulses, thereby minimizing thermal pile-up. This reduces the risk of heat-induced damage during prolonged live-cell imaging and preserves the quantitative accuracy of the measured RI change signals.

The visible probe beam is directed into a single-objective ODT system based on off-axis digital holography (DH) implemented in a Mach-Zehnder interferometer^22^. A 10-m optical fiber is incorporated into the probe-beam path to suppress temporally varying coherent artifacts. In the sample arm, the illumination angle of the visible probe is rapidly modulated using a high-speed SLM (1920 × 1152 XY Phase Series Spatial Light Modulator, Meadowlark Optics) (see “Methods”, Tilted illumination using an SLM). After transmission through the specimen, the probe beam is reflected by a silicon substrate and recollected by the same objective lens (UPlanSApo60x, Olympus), forming a reflective configuration. This geometry allows MIR excitation to be introduced from the opposite side through the substrate. The NAs for visible illumination and detection are 0.85 and 1.2, respectively. The reflected visible beam is subsequently interfered with the reference beam at an off-axis angle, and the resulting holograms are recorded at 422.4 fps using a high-speed image sensor (VLXT-17M.I, Baumer). Camera acquisition is synchronized with the SLM modulation to ensure precise angular sampling. Details of the timing synchronization scheme are provided in Supplementary Note 1.

### Validation of the spatial resolution and signal-to-noise ratio (SNR)

**Fig. 2:**
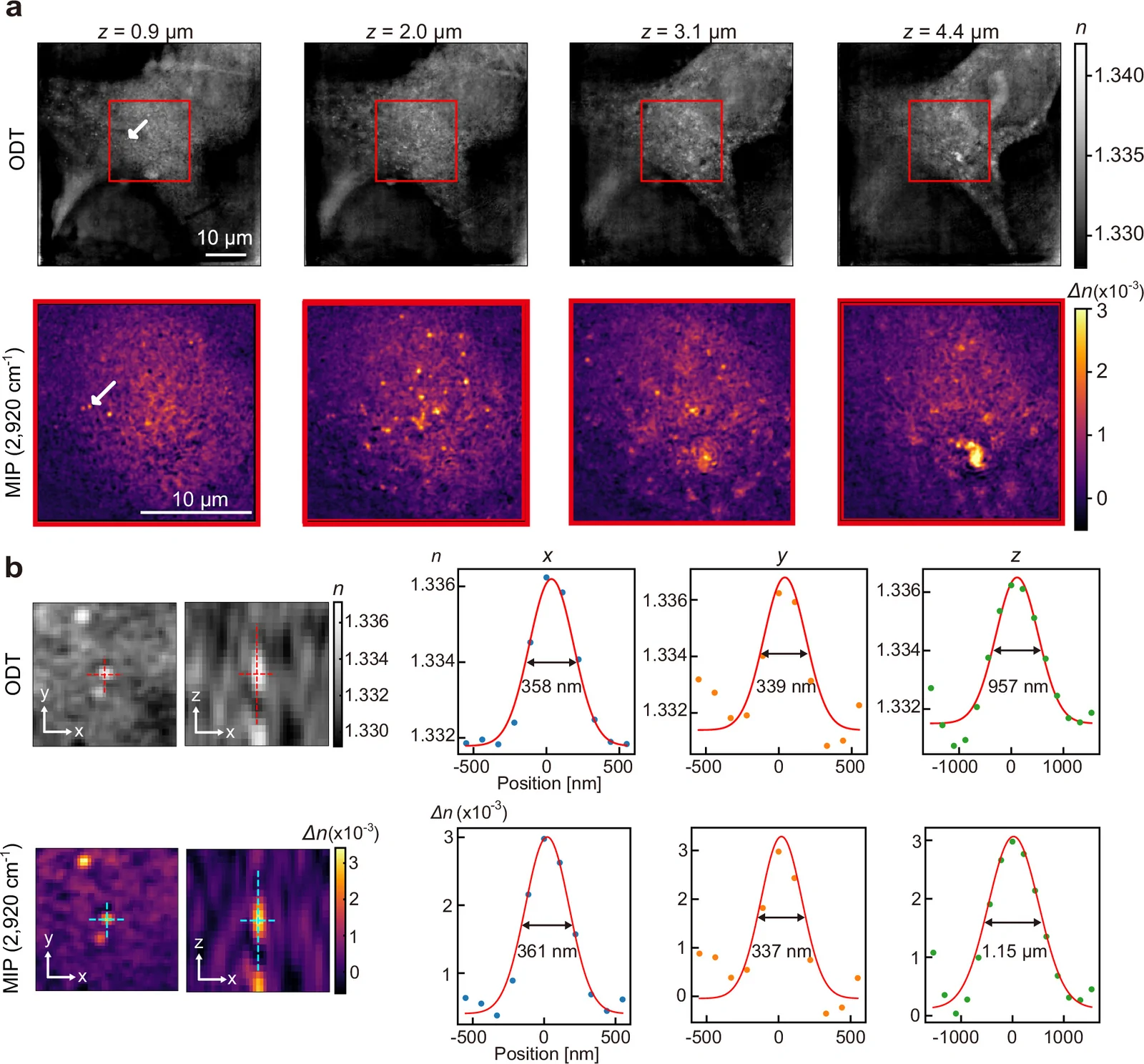
Video-rate volumetric MIP-ODT imaging and spatial resolution characterization in a living cell. **a** Depth-resolved slices extracted from the reconstructed refractive index (RI) tomogram of a living COS-7 cell (top). Corresponding MIP-induced RI change images acquired from the red-boxed regions, obtained at a volumetric imaging rate of 19.2 vps using a MIR pump tuned to 2920 cm^−^^1^ (bottom). White arrows indicate a small intracellular particle selected for spatial resolution analysis. The *z*-axis is aligned with the optical axis, and the origin is defined as the surface of the silicon substrate. The field of view (FOV) of ODT and MIP-ODT is 55 × 55 and 20 × 20 μm^2^, respectively. The sample extent of MIP-ODT was estimated to be 10.1 μm along the axial direction. ODT and MIP-ODT imaging, as shown in (**a**), was performed on more than 30 cells from more than 10 independent cultures with similar results. **b** Left, zoom-in views of the selected particle in the reconstructed RI (top) and MIP-induced RI change (bottom). Right, corresponding lateral (*x*, *y*) and axial (*z*) cross-sectional profiles extracted along the dashed lines in the left panels. Red solid lines represent Gaussian fits used to determine the full width at half maximum (FWHM). MIR mid-infrared; MIP mid-infrared photothermal; ODT optical diffraction tomography.

To validate the spatial resolution and SNR of video-rate MIP-ODT, we performed volumetric imaging of a living COS-7 cell (see “Methods”, Preparation of biological samples). Figure 2a shows representative depth-resolved slices of the RI tomograms (top) and the corresponding MIP images (bottom), acquired at a volumetric imaging rate of 19.2 vps. For MIP-ODT, a MIR pump wavenumber of 2920 cm^−^^1^ was used to excite the CH_2_ asymmetric stretching vibration, which is abundant in lipid-rich cellular structures. In both ODT and MIP-ODT, depth-resolved slices reveal plane-specific 3D cellular features that vary with axial position, confirming successful volumetric reconstruction under video-rate acquisition conditions.

We first quantified the measurement noise to determine the SNR. The temporal RI fluctuations were measured from ODT data acquired in the MIR-OFF state. Averaging the voxel-wise standard deviations across the field of view yielded an effective noise level of 4.2 × 10^−^^5^ (Supplementary Note 2). Using a representative peak MIP-induced RI change of 3.0 × 10^−^^3^ from Fig. 2a as the signal amplitude, we obtain a signal-to-noise ratio of 71.4. This value indicates that a high signal-to-noise ratio is maintained under video-rate volumetric acquisition.

Next, spatial resolution was evaluated by analyzing the full width at half maximum (FWHM) of cross-sectional profiles extracted from a small intracellular particle, as indicated by white arrows in Fig. 2a, in both the reconstructed RI and the MIP-induced RI change. Cross-sectional profiles along the lateral (*x* and *y*) and axial (*z*) directions are presented in Fig. 2b. Each profile was fitted with a Gaussian function to extract the corresponding FWHM values. For ODT, the measured FWHMs were 358, 339, and 957 nm along the *x*, *y*, and *z* axes, respectively. For MIP-ODT, the corresponding values were 361, 337 nm, and 1.15 μm. These values are taken as representative lateral and axial spatial resolutions of the present system. Numerical simulations predict a theoretical FWHM of 230 nm laterally and 870 nm axially for the point spread function. The slightly larger experimental values likely arise from the finite size of the intracellular particle used for evaluation, suggesting that the intrinsic resolution of the optical system may be modestly better than the measured estimates.

### High-speed broadband multi-wavenumber spectral volumetric imaging

We next demonstrate high-speed broadband multi-wavenumber spectral volumetric imaging by integrating the video-rate MIP-ODT platform with rapid wavenumber sweeping of the MIR excitation. This implementation enables the acquisition of up to 19.2 spectral sampling points per second within a selected spectral window. In this experiment, we focus on the 2800–3100 cm^−^^1^ region, which includes the CH_2_ and CH_3_ stretching vibrations that are characteristic of lipids and proteins. At each volume, the instantaneous MIR excitation wavenumber, corresponding to the idler output of the OPO, is determined by measuring the wavelength of the counterpart near-infrared (NIR) signal beam using a spectrometer (C11482GA, Hamamatsu). This approach allows volume-by-volume spectral assignment under fully synchronized system control (see “Methods”, Spectral volumetric analysis). Supplementary Note 3 summarizes the MIR wavenumber trajectories inferred from the measured NIR spectra for both unidirectional fast-sweeping and continuous-sweeping modes. These measurements confirm that spectral windows of approximately 300 and 200 cm^−^^1^ can be covered within one second under unidirectional and continuous sweeping modes, respectively.

**Fig. 3:**
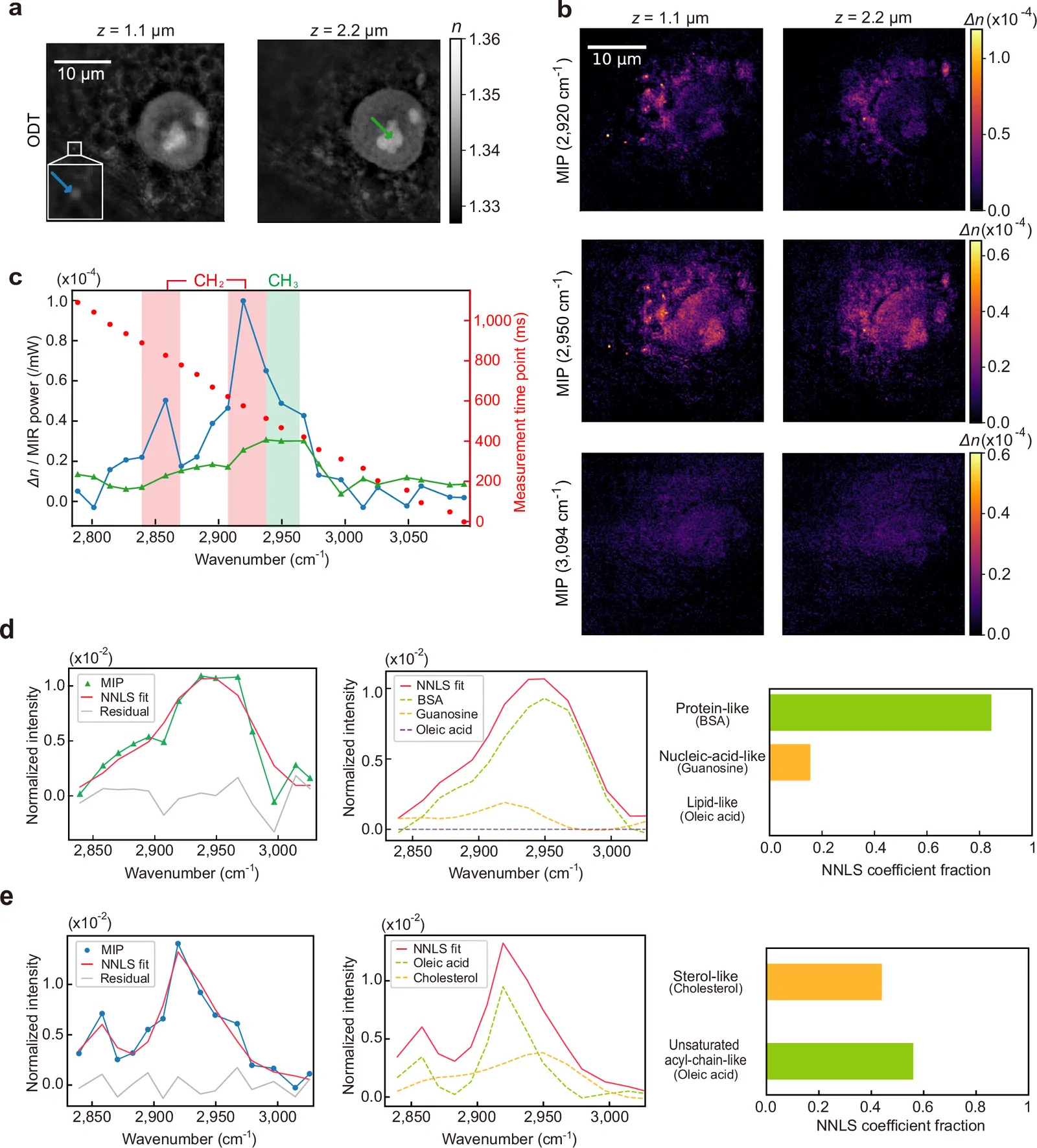
High-speed broadband multi-wavenumber spectral MIP-ODT imaging of subcellular chemical heterogeneity. **a** Representative depth-resolved slices from RI tomograms of a fixed COS-7 cell at two axial positions. The blue and green arrows indicate a small cytoplasmic particle and the nucleolus, respectively. **b** Corresponding MIP images acquired at 2920 cm^−^^1^ (top), 2950 cm^−^^1^ (middle) and 3094 cm^−^^1^ (bottom). The FOV of ODT and MIP-ODT was 44 × 44 μm^2^. The sample extent of MIP-ODT was estimated to be 2.4 μm along the axial direction. The experiments in (**a**, **b)** were independently repeated three times using three independent cell cultures (8 cells in total) with similar results. **c** Local vibrational spectra extracted from two representative subcellular regions indicated in a: the small cytoplasmic particle (blue) and the nucleolus (green). The particle and nucleolar spectra were averaged over regions of interest containing 18 and 2205 voxels, respectively. Red- and green-shaded bands indicate the CH_2_- and CH_3_-stretching regions, respectively. Red dots represent the instantaneous MIR excitation wavenumber at each measurement time point during the wavenumber sweep; the time axis denotes the elapsed time from the start of the measurement. **d**,** e** non-negative least-squares (NNLS) analysis of the nucleolar spectrum (**d**) and the cytoplasmic-particle spectrum (**e**). The nucleolar spectrum was fitted using bovine serum albumin (BSA), guanosine, and oleic acid, whereas the cytoplasmic-particle spectrum was fitted using oleic acid and cholesterol. The left panels show the measured spectra, NNLS fits, and residuals; the middle panels show the coefficient-weighted reference spectra; and the right panels show the NNLS coefficients normalized to sum to unity. MIR mid-infrared; MIP mid-infrared photothermal; ODT optical diffraction tomography.

To validate 3D, location-dependent spectral readout in cells, we performed broadband multi-wavenumber spectral volumetric imaging of a fixed COS-7 cell immersed in D_2_O-based phosphate-buffered saline (PBS). Figure 3a and b present representative depth-resolved slices of the RI tomograms and the corresponding MIP images, respectively. The MIP images shown in Fig. 3b were acquired at excitation wavenumbers of 2920, 2950 and 3094 cm^−1^. The signal at 2920 cm^−^^1^ mainly reflects CH₂ contributions that are prominent in lipid-enriched regions, whereas the 2950 cm^−^^1^ signal mainly reflects CH_3_ contributions that are relatively enhanced in protein-rich regions. Because protein-associated C–H stretching absorption forms a broad spectral band that contributes to both wavenumbers, the 2920 and 2950 cm^−^^1^ images should not be interpreted as simple lipid- and protein-specific contrasts, respectively. Nevertheless, comparison of these two resonant images reveals location-dependent spectral contrast: the cytoplasmic particle marked by the blue arrow shows a stronger signal at 2920 cm^−^^1^, whereas the nucleolus marked by the green arrow shows comparable contrast at both wavenumbers. The off-resonant image at 3094 cm^−1^ further shows that the background signal is sufficiently weak. Using the signal intensity of the blue-arrow-marked particle relative to the spatially averaged background signal in the off-resonant 3094 cm^−^^1^ image, we estimated a signal-to-background ratio of 56.

Based on the volumetric reconstructions, we selected two representative subcellular regions of interest: the nucleolus and a small cytoplasmic particle. Local vibrational spectra were extracted from these regions, as shown in Fig. 3c. The nucleolar spectrum showed a broad C–H stretching band centered near 2950 cm^−1^. To obtain a coarse molecular decomposition of this spectrum, we performed non-negative least-squares (NNLS) fitting using bovine serum albumin (BSA), guanosine, and oleic acid as representative references for protein-like, nucleic-acid-like, and lipid-like spectral contributions, respectively (Fig. 3d) (see “Methods”, Reference-based NNLS analysis of local C–H stretching spectra). The fitted spectrum closely reproduced the measured MIP spectrum with a small residual (Fig. 3d, left), and the fitted contributions of the three reference components are shown in Fig. 3d, middle. The normalized NNLS coefficients showed a dominant protein-like contribution, a secondary nucleic-acid-like contribution, and only a minor lipid-like contribution (Fig. 3d, right). This decomposition is consistent with the expected protein- and RNA-dominant molecular composition of the nucleolus.

The spectrum extracted from the small cytoplasmic particle exhibited pronounced CH₂-associated peaks around 2860 and 2920 cm^−1^, consistent with a lipid-rich compartment and supporting its assignment as a lipid droplet. We then examined its lipid-related spectral composition by two-component NNLS fitting using oleic acid and cholesterol as representative references for unsaturated acyl-chain-like and sterol-like spectral contributions, respectively (Fig. 3e). The fitted spectrum closely reproduced the measured MIP spectrum with a small residual (Fig. 3e, left), and the fitted contributions of the two reference components are shown in Fig. 3e, middle. The normalized NNLS coefficients indicated that the unsaturated acyl-chain-like component accounted for approximately 56% of the fitted spectral contribution, whereas the sterol-like component accounted for approximately 44% (Fig. 3e, right).

The larger unsaturated-acyl-chain-like contribution is qualitatively consistent with the neutral-lipid-rich composition of lipid droplets, whose cores commonly contain triacylglycerols and sterol esters with fatty acyl chains^23^. These fractions represent relative spectral weights within the present reference-based model and should not be interpreted as quantitative molar fractions of the underlying lipid species. The ability to perform local lipid-composition analysis in three dimensions with approximately second-scale temporal resolution could support future live-cell studies that link lipid-droplet composition to droplet transport, organelle interactions, and longer-term metabolic remodeling. These results show that broadband multi-wavenumber spectral MIP-ODT resolves compartment-specific vibrational signatures in 3D and enables rapid chemical phenotyping of intracellular architecture.

### Video-rate 3D tracking of lipid droplets in living cells

To demonstrate a biologically relevant use case of video-rate MIP-ODT, we performed 3D tracking of lipid droplets in living cells. Lipid droplets are key targets in label-free vibrational imaging because their spatial distribution, intracellular transport, and structural remodeling reflect cellular metabolism and interactions with organelles, the cytoskeleton, and local intracellular environments. Video-rate MIP-ODT enables these lipid-rich structures to be visualized and tracked in three dimensions without labeling.

**Fig. 4:**
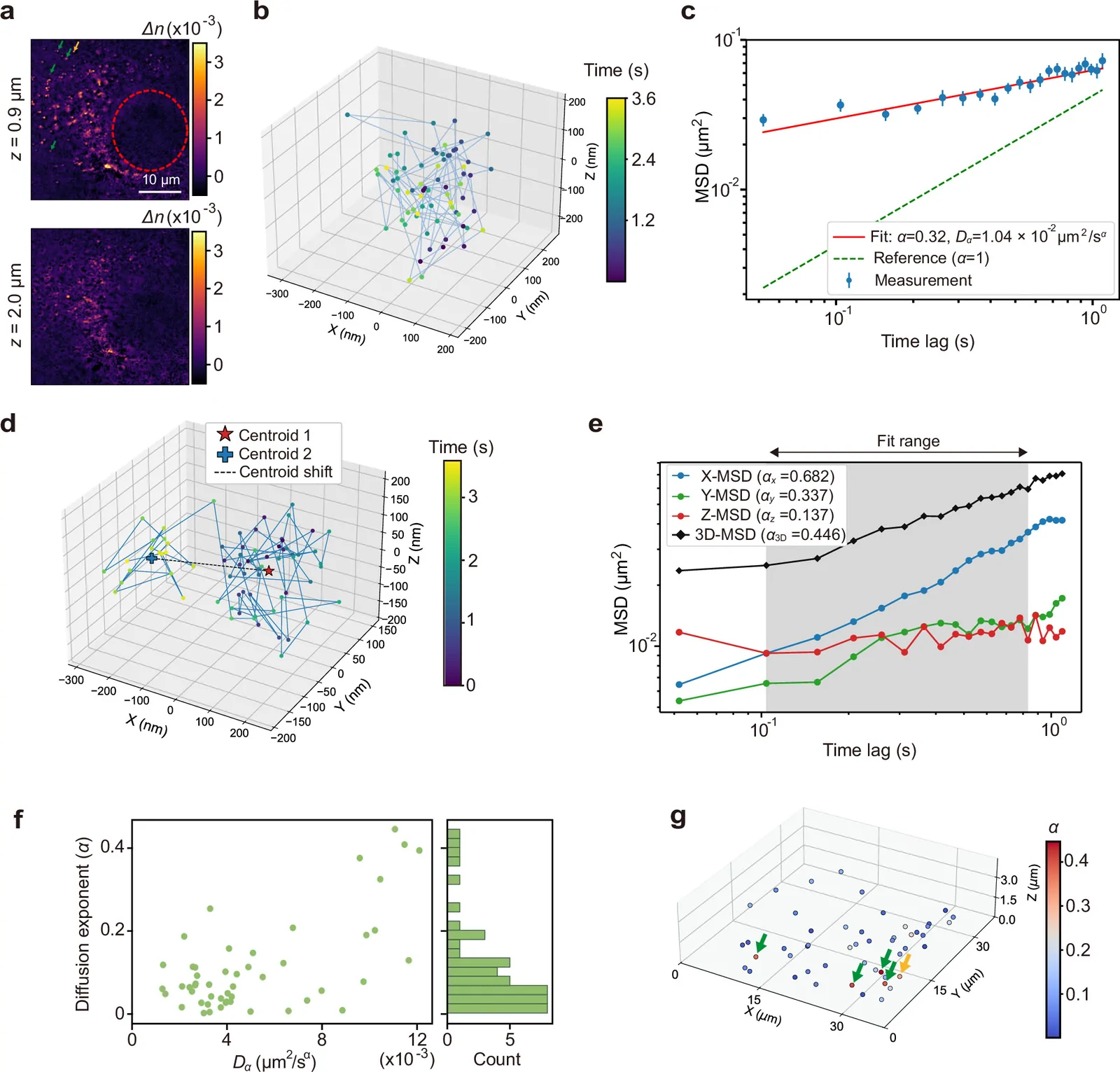
Video-rate 3D tracking and anomalous-diffusion analysis of lipid droplets in living cells. **a** Representative depth-resolved MIP-ODT slice of an oleic-acid-treated COS-7 cell acquired at 19.2 volumes per second with MIR excitation tuned to 2920 cm^−1^. The red dashed circle indicates the nuclear region. The orange arrow indicates the lipid droplet analyzed in (**b**, **c**), the red arrow indicates the lipid droplet analyzed in (**d**, **e**), and the green arrows indicate additional lipid droplets highlighted in (**g**). The FOV of MIP-ODT is 37.4 × 37.4 μm^2^. The sample extent of MIP-ODT was estimated to be 6.4 μm along the axial direction. The video-rate measurement in (**a**) was performed on three cells in different fields of view from a single dish, with similar results. **b** Reconstructed 3D trajectory of a lipid droplet indicated by the orange arrow in (**a**), tracked over a 3.6 s acquisition window. **c** Mean square displacement (MSD) calculated from the 3D trajectory as a function of time lag τ. **d** The reconstructed 3D trajectory of the lipid droplet indicated by the red arrow in (**a**), exhibiting a step-like displacement between two spatially separated regions. The red star and blue cross indicate the trajectory centroids calculated before and after the displacement, respectively, and the dashed line indicates the centroid-to-centroid displacement. **e** Direction-resolved MSDs along the *x, y, z* axes, together with the full 3D MSD, calculated from the trajectory shown in (**d**). The gray shaded region and the arrow indicate the range of time lags used for power-law fitting. **f** Scatter plot of the generalized diffusion coefficient ${D}_{\alpha }$ versus the anomalous diffusion exponent α obtained from MSD analysis of individual lipid-droplet trajectories in the field of view. The histogram on the right shows the distribution of α across trajectories (counts per bin). **g** Spatial map of anomalous diffusion exponents α. The orange, red and green arrows identify the lipid droplets marked by the corresponding arrows in a.

We performed volumetric MIP-ODT imaging of living COS-7 cells treated with oleic acid, a condition known to robustly induce lipid droplet formation^24^. Volumetric datasets were acquired continuously for approximately 3.6 s at 19.2 vps, with the MIR excitation tuned to 2920 cm^−1^ to selectively probe lipid-rich structures. Representative depth-resolved slices are shown in Fig. 4a. Using this dataset, we analyzed the motion of an individual lipid droplet exhibiting pronounced displacement during the acquisition period, as indicated by the orange arrow (see “Methods”, Motion analysis in video-rate MIP-ODT imaging). The centroid position was tracked over time to reconstruct its full 3D trajectory (Fig. 4b). The tracking results overlaid on the MIP-ODT data are shown in Supplementary Movie 1. The displacement spans approximately 200–300 nm along each of the *x*, *y*, and *z* axes. Such axial motion cannot be captured by conventional 2D imaging and would therefore lead to systematic underestimation of intracellular transport dynamics. In contrast, our volumetric imaging enables direct quantification of complete 3D motion within the cellular environment. 3D tracking of lipid droplets has previously been reported using ODT^25^, in which droplets were identified from high-RI puncta. However, this contrast mechanism lacks intrinsic chemical specificity and may therefore require orthogonal validation. By contrast, MIP-ODT directly tracks lipid-rich structures via vibrational contrast, enabling chemically specific and volumetrically resolved characterization of droplet dynamics.

To quantify the biological information provided by 3D tracking, we analyzed the centroid trajectory within the framework of anomalous diffusion. This analysis is valuable because anomalous diffusion reflects constrained intracellular transport arising from molecular crowding, viscoelasticity, and interactions with local intracellular environments. The mean squared displacement (MSD), calculated from the 3D trajectory over the 3.6 s observation window and evaluated for time lags τ up to 1.1 s, exhibits a power-law dependence on *τ* (Fig. 4c). Fitting the MSD yields an anomalous diffusion exponent of *α* = 0.32 (95% bootstrap confidence interval (CI): 0.23–0.42, 500 bootstrap resamples), which is substantially lower than the value expected for free diffusion (*α* = 1) and indicates pronounced subdiffusive behavior. Such subdiffusion is commonly observed for endogenous cargos in living cells and is generally attributed to molecular crowding and viscoelastic constraints that impede long-range transport^26^.

To illustrate the information uniquely provided by volumetric tracking, we next analyzed a distinct lipid droplet, indicated by the red arrow in Fig. 4a, whose trajectory exhibited a step-like change in its local mean position during the observation window. Segmenting the trajectory before and after the transition revealed two spatially separated centroid positions, with a three-dimensional centroid shift of approximately 0.28 µm (Fig. 4d). The trajectory therefore contained both local fluctuations around transient centroid positions and a larger relocation between them, a behavior that can be fully quantified only when the particle position is measured in *x*, *y*, and *z* at each time point. We further decomposed the MSD of this trajectory into Cartesian components (Fig. 4e). The fitted anomalous exponents differed substantially among axes, with ${\alpha }_{X}$ = 0.68, ${\alpha }_{Y}$ = 0.34, ${\alpha }_{Z}$ = 0.14, and ${\alpha }_{3{{{\rm{D}}}}}$ = 0.45 for the total 3D MSD over the same fit range. This axis-resolved analysis reveals direction-dependent intracellular constraints that would be concealed in a 2D projection, in which axial confinement and its contribution to the overall transport mode cannot be evaluated. These results underscore that video-rate MIP-ODT enables not only chemically specific tracking of lipid droplets but also quantitative decomposition of their 3D transport dynamics.

We next estimated the generalized diffusion coefficient ${D}_{\alpha }$ and the diffusion exponent α for 50 tracked particles within the same field of view. Figure 4f summarizes the resulting distributions of ${D}_{\alpha }$ and α. The particle-wise estimation uncertainty was moderate (median 95% bootstrap CI width for α: 0.17, median relative 95% bootstrap CI width for ${D}_{\alpha }$: 0.17, 500 bootstrap resamples). The diffusion coefficients span approximately 10^−3^–10^−2^ µm^2^ s^−*α*^, revealing marked heterogeneity in intracellular lipid droplet mobility. A previous study has reported anomalous diffusion exponents for intracellular lipid droplets spanning a broad range from approximately 0 to 2^25^. In contrast, the *α* values observed here are confined to a narrower range of 0.0–0.4. This restricted distribution may reflect the relatively low metabolic activity of the cells under our experimental conditions, which would suppress ATP-dependent active processes, thereby biasing subcellular motion toward more locally constrained dynamics^27^.

Finally, to probe the spatial organization of intracellular mobility, we mapped the spatial distribution of diffusion exponents α across the field of view (Fig. 4g). A subset of lipid droplets marked by orange, red, and green arrows in Fig. 4a, g exhibited locally elevated α values, revealing pronounced spatial heterogeneity in diffusion behavior within the cell. These particles are located distal to the nucleus (dashed red circle in Fig. 4a), where intracellular crowding is reduced relative to perinuclear regions enriched in endoplasmic reticulum and other organelles. These observations suggest that lipid droplets experience less constrained motion in these areas. Collectively, these results demonstrate that video-rate MIP-ODT enables real-time, 3D tracking of chemically resolved intracellular structures, providing quantitative access to heterogeneous and potentially anisotropic particle dynamics in living cells.

## Discussion

### Technical scope and practical imaging regime

The present work is neither simply high-speed ODT with MIR excitation added, nor simply synthetic-aperture MIP-QPI operated at a higher angular-scanning speed. Although high-speed ODT^28^ and synthetic-aperture MIP-QPI^22^ have each been demonstrated previously, realizing high-speed MIP-ODT requires addressing additional challenges specific to MIP-based tomography. The central challenge is that weak MIP-induced phase changes must be measured with high phase stability while the illumination angle is rapidly scanned. This requires synchronized single-pulse visible probing with enough photons for sensitive phase imaging, suppression of SLM AC-drive-related noise, and reduction of temporally varying coherent artifacts that would otherwise contaminate MIR-ON/MIR-OFF differential phase measurements. In addition, direct differential tomographic reconstruction is required to recover volumetric MIP-induced RI changes while minimizing reconstruction artifacts arising from interferometer fluctuations. Together, these advances allowed us to achieve video-rate MIP-ODT for volumetric reconstruction of weak MIP-induced RI changes.

It is also important to clarify the practical regime in which this capability is demonstrated. In the present experiments, we demonstrate high-speed volumetric MIP imaging at the single-cell scale, with sample thicknesses ranging from several micrometers to approximately ten micrometers. The method may also be applicable to thin specimens with thicknesses of up to several tens of micrometers; however, extension to substantially thicker specimens or tissue-scale imaging would require additional considerations. In such regimes, strong MIR absorption by water and visible-light scattering can limit the penetration depth, while the breakdown of the weak-scattering tomographic model can limit quantitative reconstruction. Thus, the present platform is best positioned for high-resolution volumetric chemical imaging of single cells and thin biological specimens, rather than deep tissue-scale imaging.

### Future technical development of MIP-ODT

We next discuss several directions for further technical development of MIP-ODT. A central opportunity is extending SLM-based wavefront control toward fully programmable illumination. In the current implementation, video-rate volumetric imaging employs a fixed set of eleven illumination angles. Programmable illumination would enable adaptive control of angular sampling density according to the characteristic timescale of the dynamics under investigation. This flexibility provides a tunable trade-off between reconstruction fidelity and acquisition speed, as illustrated in Fig. 1b. For example, when observing biological processes evolving on second timescales, the number of illumination angles can be increased by approximately an order of magnitude, thereby achieving reconstruction fidelity comparable to that obtained with 60 illumination angles in Fig. 1b, provided that strict video-rate acquisition is not required.

Programmable illumination also enables more advanced acquisition strategies. The SLM can generate complex illumination patterns that implement angular multiplexing within a single camera exposure, in combination with an appropriate forward model and model-based inversion^28^. Encoding multiple illumination angles into a single measurement expands Fourier-space coverage per exposure, thereby improving the fidelity of reconstructed MIP-induced RI-changes while preserving the volumetric imaging rate. Alternatively, angular multiplexing can maintain the same equivalent angular density while reducing the total number of measurement frames. This strategy enables several-fold increases in volumetric acquisition speed without compromising reconstruction fidelity.

We further consider resolution enhancement enabled by the reflective ODT geometry adopted here. Compared with conventional transmission-based implementations, this geometry provides access to higher illumination NAs^22^. In the present experiment, however, the ODT resolution was mainly limited by the modest illumination NA, which was chosen to balance axial resolution, lateral field of view, and observable depth range for single-cell volumetric imaging. If spatial resolution is prioritized, increasing the illumination and collection NAs to 1.2 and 1.3 is expected to improve spatial resolution to 168 nm laterally and 242 nm axially. Additional improvement can be achieved by shortening the probe wavelength. A 400 nm probe would yield estimated resolutions of 126 nm laterally and 181 nm axially, enabling 3D resolution of sub-200-nm structures. This resolution regime would permit label-free visualization of intracellular nanostructures that remain challenging for conventional 3D chemical imaging, including small vesicular compartments and membrane-associated nanostructures.

We also consider the extension of the accessible spectral range. In the present implementation, the MIR wavelength range was limited by a nanosecond OPO based on a fan-out PPLN crystal, which restricted the output to the high-wavenumber region. This limitation originates from the specific nonlinear crystal used in the current OPO, rather than from the MIP-ODT platform itself. Fingerprint-region MIP-ODT should therefore be feasible using a suitable nanosecond MIR source based on nonlinear crystals such as AGS^29^ or BGSe^30^. This extension would broaden the chemical specificity of the video-rate MIP-ODT platform by enabling access to fingerprint vibrational bands.

### Future applications in dynamic intracellular chemical imaging

This platform enables chemically specific 3D imaging of intracellular dynamics over sub-second to extended observation windows. One promising application is the study of lipid droplets, whose formation, transport, and remodeling are closely linked to cellular metabolism. Extending the present analysis of lipid-droplet motion to longer observation windows could capture a broader sequence of dynamic processes, including de novo lipid-droplet formation from the endoplasmic reticulum, growth following fatty-acid loading, fusion, fragmentation, redistribution, and responses to metabolic remodeling or cellular stress^23^. Volumetric tracking could also reveal how lipid droplets interact with organelles, cytoskeletal networks, and local intracellular environments during these processes. Combined with broadband spectral acquisition, this framework could enable future time-lapse studies that relate 3D lipid-droplet transport to local compositional changes, extending the local spectral decomposition demonstrated in Fig. 3.

High-speed 3D chemical imaging could also provide a powerful approach for studying biomolecular condensates formed through liquid-liquid phase separation, including nucleolar reorganization and stress-induced assemblies whose molecular components exchange on timescales of seconds to minutes^31^. In this context, broadband multi-wavenumber spectral MIP-ODT is particularly attractive because it can acquire a wavelength-swept volumetric dataset in approximately 1 s. This capability could capture both the 3D morphological evolution of condensates, such as nucleation, growth, fusion, dissolution, and coarsening, and accompanying changes in local chemical composition. With access to appropriate vibrational bands, spectral MIP-ODT could further reveal spatially heterogeneous molecular remodeling within condensates, including changes in protein-rich or nucleic-acid-rich regions, hydration state, molecular packing, and spectral signatures associated with protein secondary structure during condensate maturation or stress responses. These examples position MIP-ODT as a versatile platform for high-speed, label-free volumetric chemical imaging and for quantitative analysis of dynamic intracellular organization and transport.

## Methods

### Procedure of RI reconstruction

3D RI reconstruction was performed using the standard ODT workflow under the Rytov approximation^32^. The MIP-ODT reconstruction workflow was modified relative to conventional approaches. In conventional implementations, RI distributions are independently reconstructed for the MIR-ON and MIR-OFF states from complex optical fields referenced to background images measured in cell-free regions. The MIP-induced RI change is then obtained by subtracting the reconstructed MIR-OFF distribution from the MIR-ON distribution.

In this study, we introduced a direct differential reconstruction method that directly estimates the MIP-induced RI change from the differential complex optical fields between the MIR-ON and MIR-OFF states. This strategy removes the need for separate background acquisition in cell-free regions, thereby reducing experimental overhead and supporting real-time MIP imaging. It also suppresses reconstruction artifacts caused by interferometer fluctuations. In practice, the MIR-ON and MIR-OFF phase images can contain small phase offsets and slowly varying phase gradients that are unrelated to the photothermal signal. These residual phase errors mainly arise from interferometer fluctuations caused by mechanical instability and air-current disturbances. Because the MIP-induced RI change is small, these residual phase errors can become comparable to or larger than the true MIP signal. In the conventional reconstruction-then-subtraction approach, such errors can propagate through the two separate 3D reconstructions and appear as non-photothermal artifacts in the final subtracted volume. Direct differential reconstruction avoids this problem by correcting offset and gradient errors before 3D reconstruction, thereby preventing such artifacts from propagating into the reconstructed MIP volume. A detailed formulation of the reconstruction method is provided in Supplementary Note 4.

In our reflection-mode ODT configuration, the visible probe beam interacts with the sample during both the incident and substrate-reflected paths. This double-pass geometry can generate a mirror-image contribution relative to the silicon substrate. In the reconstructed 3D volume, however, this contribution appears at an axial position distinct from the actual sample volume and can therefore be separated from the true object (Supplementary Note 5). Accordingly, the subsequent analysis was performed using cropped subvolumes containing the actual sample volume while excluding the mirror-image contribution.

After this separation, the ODT and MIP-ODT tomograms used for analysis span (X) 58.7 × (Y) 58.7 × (Z) 29.3 μm^3^. To focus on the MIR-irradiated region, we further analyzed cropped subvolumes for each experiment. The cropped volumes are reported in the corresponding figure captions. The sample extent was estimated as the apparent axial range over which the lipid droplet contrast was observable, measured from the silicon substrate surface.

### ODT measurement with a variable number of illuminations

To evaluate the effect of angular sampling density on reconstruction performance, we performed ODT measurements using a variable number of illumination angles, shown in Fig. 1b. Although this dataset was acquired using an optical configuration that differs from that in Fig. 1e, these modifications do not affect the conclusion of this analysis. In this configuration, a 1 ns visible pulse source was employed instead of the 10 ns laser used in Fig. 1e. In addition, the SLM was replaced with a wedge prism mounted on a rotation stage. The illumination NA was fixed at 1.0. Controlled azimuthal rotation of the prism enabled precise tuning of the illumination angle while maintaining a constant illumination NA.

### Tilted illumination using an SLM

Tilted illumination was generated using an SLM programmed with a blazed phase grating pattern. This grating efficiently directs the incident optical power into the first diffraction order, which was used as the tilted illumination beam. To suppress undesired diffraction components, including the zeroth order and higher orders, spatial filtering was applied using an aperture mask placed at a Fourier plane. As a result, only the desired coherent tilted illumination was delivered to the specimen, enabling controlled angular illumination for volumetric imaging (Supplementary Note 6).

Because the SLM is operated with an alternating-current (AC) voltage, small periodic phase fluctuations are inherently introduced into the modulated wavefront. To mitigate the resulting phase noise, the timing of the MIR pump pulses, the visible probe pulses, and the camera exposure was synchronized to the AC driving cycle of the SLM.

### Preparation of biological samples

COS-7 cells (JCRB9127, JCRB Cell Bank) are cultured on a silicon substrate with a thickness of 675 μm (6-675P1UMM, AKD, Japan). Cells were maintained in high-glucose Dulbecco’s modified Eagle medium (DMEM; FUJIFILM Wako) containing L-glutamine, phenol red, and HEPES, supplemented with 10% fetal bovine serum (FBS; Cosmo Bio) and 1% penicillin-streptomycin-L-glutamine solution (FUJIFILM Wako). Cell cultures were maintained at 37 °C in a humidified incubator with 5% CO_2_.

Prior to imaging, samples were mounted by placing a glass coverslip directly on top of the cells cultured on the silicon substrate. For the experiment shown in Fig. 3, cells were chemically fixed with 4% paraformaldehyde, after which the culture medium was replaced with D_2_O-based PBS for imaging. For the live-cell experiment shown in Fig. 4, lipid-enriched cells were prepared by supplementing the culture medium with 100–200 μM of oleic acid and bovine serum albumin complex, followed by overnight incubation.

### Spectral volumetric analysis

Broadband multi-wavenumber spectral MIP-ODT datasets were acquired by synchronizing volumetric image acquisition with the spectrometer readout using an index-based pairing scheme. A detailed description of the timing chart is provided in Supplementary Fig. 6a in Supplementary Note 7. At the beginning of the measurement, the MIR beam was blocked using a mechanical shutter, and baseline MIR-OFF signals were recorded by both the camera and the spectrometer. After a short delay, the shutter was opened, producing a step-like increase in the MIR-induced signal. This shutter-opening time point was identified during post-processing and defined as the temporal origin of the spectral measurement. Volumetric datasets acquired after the temporal origin were paired with the corresponding spectrometer records by matching their sequential acquisition indices.

The MIR idler wavenumber associated with each volume was calculated from the measured NIR signal wavenumber using the energy conservation relation of the OPO, ${\omega }_{{{{\rm{pump}}}}}={\omega }_{{{{\rm{signal}}}}}+{\omega }_{{{{\rm{idler}}}}}$. To correct for wavenumber-dependent variations in MIR excitation power during spectral reconstruction, each MIP-ODT volume was normalized by the measured MIR pulse energy at the corresponding wavenumber. Because the MIR wavenumber is scanned continuously during each volumetric acquisition, the MIR pulse energy can vary slightly even within a single volume. Over the scanned spectral range, however, the MIR power dependence on wavenumber is smooth, and the resulting intra-volume variation is limited to at most 4.6% in relative intensity (Supplementary Fig. 6b). We therefore expect spectral distortion introduced by this normalization to be negligible.

### Reference-based NNLS analysis of local C-H stretching spectra

Local MIP spectra were obtained by averaging the signal within three-dimensional regions of interest (ROIs) in the reconstructed volumes and assigning each volume to the corresponding MIR excitation wavenumber. The spectra were cropped to 2800–3100 cm^−1^, baseline-corrected using asymmetric least squares (*λ* = 10^5^, *P* = 0.01, 20 iterations), and area-normalized over 2830–3027 cm^−1^.

Reference-based spectral decomposition was performed over 2830–3027 cm^−1^ using constrained linear least-squares fitting. Non-negativity constraints were imposed on the reference-spectrum coefficients, whereas the slope and intercept of an additional linear baseline term were left unconstrained. The nucleolar spectrum was fitted using BSA, guanosine, and oleic acid as representative references for protein-like, nucleic-acid-like, and lipid/acyl-chain-like contributions, respectively. The spectrum of the small cytoplasmic particle was fitted using oleic acid and cholesterol as representative references for unsaturated-acyl-chain-like and sterol-like contributions, respectively.

The BSA reference spectrum was measured by FTIR in D_2_O at a nominal spectral resolution of 16 cm^−1^. Reference spectra of guanosine, oleic acid, and cholesterol were obtained from the NIST Chemistry WebBook and were converted from transmittance to absorbance where necessary. The NIST reference spectra were Gaussian-broadened to an effective spectral resolution of 16 cm^−1^, matching the nominal resolution of the BSA spectrum and closely approximating the effective spectral resolution of the MIP measurements. The BSA spectrum was used without further broadening. All reference spectra were then linearly interpolated onto the experimental wavenumber grid.

Because uncertainty in the timing between MIP acquisition and spectrometer-based wavenumber readout can introduce a small offset in the absolute wavenumber axis, it was corrected using the reference spectra. The global wavenumber shift used for this correction was determined by grid search to minimize the combined residual sum of squares for the nucleolar and small-particle fits and was then applied uniformly to all measured spectra. The fitted reference-spectrum coefficients were normalized by their sum and are reported as relative spectral weights rather than molecular fractions or absolute concentrations. Fitting was performed in Python using SciPy’s scipy.optimize.lsq_linear function.

### Motion analysis in video-rate MIP-ODT imaging

Lipid droplets were detected and tracked using a supervised image-analysis pipeline. Lipid-droplet candidates were identified by the Pixel Classification in the ilastik library^33^. The classifier was trained on manually curated in-house annotations that included only well-isolated droplets. Because reliable MSD analysis requires non-overlapping single-particle trajectories, droplets located in densely packed or clustered regions were deliberately excluded from the training set. As a result, the detection pipeline preferentially identifies spatially separated droplets and may under-represent droplets in high-density regions. For each reconstructed 3D volume, the lipid-droplet probability channel was extracted from the ilastik output and thresholded at *P* ≥ 0.8. The thresholded volumes were subjected to 3D connected-component labeling. Components smaller than 5 voxels were discarded. Particle positions were defined as probability-weighted centroids of the remaining components. Trajectory reconstruction was performed using the trackpy library^34^. Tracking parameters were empirically optimized to suppress false detections and incorrect linking events. The maximum allowable inter-frame displacement was limited to 0.5 μm. Trajectories shorter than 60 frames out of a total of 70 frames were excluded to ensure robust MSD estimation.

For each retained trajectory, the centroid position at frame i was represented as ${{{{\bf{r}}}}}_{i}=({x}_{i},\,{y}_{i},\,{z}_{i})$. The axis-resolved time-averaged MSD was calculated as ${{{\rm{MS}}}}{{{{\rm{D}}}}}_{q}\left({\tau }_{m}\right)=\left\langle {\left|q\left(t+{\tau }_{m}\right)-q\left(t\right)\right|}^{2}\right\rangle,{q}\in \left\{x,{y},{z}\right\}$, where $\langle \cdot \rangle$ denotes a time average over all valid time points t. The total 3D MSD was calculated as ${{{\rm{MS}}}}{{{{\rm{D}}}}}_{3{{{\rm{D}}}}}\left(\tau \right)=\,\left\langle {\left|{{{\bf{r}}}}\left(t+\tau \right)-{{{\bf{r}}}}\left(t\right)\right|}^{2}\right\rangle={{{\rm{MS}}}}{{{{\rm{D}}}}}_{x}\left(\tau \right)+{{{\rm{MS}}}}{{{{\rm{D}}}}}_{y}\left(\tau \right)+{{{\rm{MS}}}}{{{{\rm{D}}}}}_{z}(\tau )$. To reduce bias arising from the finite trajectory length and ensure statistical robustness, the lag time τ was restricted to 30% of the total acquisition duration. The axis resolved MSDs were fitted independently using ${{{\rm{MS}}}}{{{{\rm{D}}}}}_{q}\left(\tau \right)={A}_{q}{\tau }^{{\alpha }_{q}},{q}\in \{x,{y},{z}\}$, where ${\alpha }_{q}$ is the axis-specific scaling exponent and ${A}_{q}$ is the corresponding prefactor. The prefactor was equivalently expressed as ${A}_{q}=2{D}_{{\alpha }_{q},q},$ where ${D}_{{\alpha }_{q},q}$ is the axis-specific generalized diffusion coefficient, with units of ${{{\rm{\mu }}}}{{{{\rm{m}}}}}^{2}{{{{\rm{s}}}}}^{-{\alpha }_{q}}$. The total 3D MSD was fitted independently using ${{{\rm{MS}}}}{{{{\rm{D}}}}}_{3{{{\rm{D}}}}}\left(\tau \right)=\,6{D}_{\alpha }{\tau }^{{\alpha }_{3{{{\rm{D}}}}}}$, where ${\alpha }_{3{{{\rm{D}}}}}$ is the 3D anomalous diffusion exponent and ${D}_{\alpha }$ is the generalized 3D diffusion coefficient, with the units of ${{{\rm{\mu }}}}{{{{\rm{m}}}}}^{2}{{{{\rm{s}}}}}^{-{\alpha }_{3{{{\rm{D}}}}}}$. The fitting parameters were estimated by linear regression of log(MSD) versus log(τ) over the fitting range of 10–80% of the computed lag-time. Parameter uncertainty was quantified using 95% confidence intervals derived from case bootstrap resampling of the (log(τ), log(MSD)) pairs within the fitting range. For each trajectory, 500 bootstrap resamples were generated, and the regression was refitted for each resample. Confidence intervals for the anomalous exponent α and generalized diffusion coefficient ${D}_{\alpha }$ were obtained from the corresponding percentile distributions.

For the trajectory exhibiting a step-like change in local mean position in Fig. 4d, the trajectory was divided into pre-transition and post-transition segments. The split point was selected from all candidates that retained at least five positions in each segment and was defined as the point that maximized the 3D distance between the two segment centroids. This procedure identified the boundary between frames 52 and 53. For each segment s, the local mean centroid was calculated as ${\bar{{{{\bf{r}}}}}}_{s}=\frac{1}{{N}_{s}}{\sum}_{i\in s}{{{{\bf{r}}}}}_{i},$ where ${N}_{s}$ is the number of positions in segment s. The magnitude of the 3D centroid shift was defined as $\Delta {r}_{{{{\rm{shift}}}}}=|\left|{\bar{{{{\bf{r}}}}}}_{{{{\rm{post}}}}}-{\bar{{{{\bf{r}}}}}}_{{{{\rm{pre}}}}}\right||,$ yielding a shift of approximately 0.28 μm. All 70 frames were retained, with frames 0–52 assigned to the pre-transition segment and frames 53–69 assigned to the post-transition segment. The segmentation was used only to quantify the shift between the two local mean positions. The MSDs shown in Fig. 4e were calculated from the full trajectory, including the step-like relocation. The fitted exponents therefore reflect the combined contributions of local positional fluctuations and the relocation and are interpreted as effective scaling exponents for the trajectory as a whole, rather than as anomalous diffusion exponents of a single stationary process.

In Fig. 4g, the anomalous diffusion exponent estimated for each trajectory was spatially mapped to the particle’s initial position, defined as its location in the first acquired frame.

### Statistics and reproducibility

No statistical method was used to predetermine sample size. The ODT and MIP-ODT imaging experiments corresponding to Fig. 2 were performed on more than 30 cells from more than 10 independent cultures, with similar results. The broadband multi-wavenumber imaging experiments corresponding to Fig. 3 were independently repeated three times using three independent cell cultures (8 cells in total), with similar results. The video-rate live-cell imaging experiment corresponding to Fig. 4 was performed on three cells in different fields of view from a single dish, with similar results. For the particle-tracking analysis in Fig. 4, 50 trajectories were analyzed according to the inclusion criteria and statistical procedures described in Methods, Motion analysis in video-rate MIP-ODT imaging.

### Reporting summary

Further information on research design is available in the Nature Portfolio Reporting Summary linked to this article.

## Supplementary information

Supplementary Information

Description of Additional Supplementary Files

Supplementary Movie 1

Reporting Summary

Transparent Peer Review file

## Source data

Source Data

## Data Availability

The raw microscopy datasets generated in this study and related spectroscopic data have been deposited in Zenodo https://doi.org/10.5281/zenodo.22685837^35^. All source data underlying the figures in this paper are provided in the Source Data file. Source data are provided with this paper.

## Code availability

The images were acquired using Baumer Camera Explorer v3.4.4. Custom analysis code used to generate all figures is available on GitHub (https://github.com/Ideguchi-Lab/Video-rate-volumetric-chemical-imaging-via-mid-infrared-photothermal-optical-diffraction-tomography) and archived in Zenodo (v1.0.0; https://doi.org/10.5281/zenodo.22685210^36^). The full list of dependent modules is available in the uv.lock file.
