# Supplementary material for: Video-rate volumetric chemical imaging via mid-infrared photothermal optical diffraction tomography
Source: Nat Commun. 2026 Oct 30;17:11075. doi: 10.1038/s41467-026-78699-z (PMC13722699; doi:10.1038/s41467-026-78699-z)
Supplement: Supplementary file 1 [file 41467_2026_78699_MOESM1_ESM.pdf]

Supplementary Information for
Video-rate volumetric chemical imaging via mid-infrared photothermal optical diffraction tomography

Masato Fukushima[1], Keiichiro Toda[2*], Yusei Sugawara[1], Shotaro Kawano[2], and Takuro Ideguchi[1,2,3,4**]

[1] Department of Physics, The University of Tokyo, Tokyo, Japan

[2] Institute for Photon Science and Technology, The University of Tokyo, Tokyo, Japan

[3] Department of Advanced Materials Science, The University of Tokyo, Chiba, Japan

[4] RIKEN Center for Advanced Photonics, RIKEN, Japan

[*] keiichiro--toda@g.ecc.u-tokyo.ac.jp

[**] ideguchi@g.ecc.u-tokyo.ac.jp

Supplementary Note 1: Synchronization system for video-rate MIP-ODT.
Supplementary Figure 1 illustrates the synchronization architecture for video-rate MIP-ODT operation. A function generator provides a master TTL clock at 422.4 Hz, which serves as the timing reference for the entire system. This clock is distributed to the Q-switched laser that generates the MIR pump pulse, a delay generator, an optical chopper controller, and an Arduino-based timing controller to ensure deterministic synchronization across all components.

The delay generator triggers a second Q-switched laser that produces the visible probe pulse. The delay is set to 3-4 ns, allowing the probe pulse to capture the photothermal response before substantial thermal diffusion occurs. MIR excitation is intensity-modulated at 19.2 Hz using an optical chopper phase-locked to the master clock. Within each volumetric acquisition cycle, the MIR state alternates every 11 pulses, forming consecutive 11-pulse MIR-OFF and 11-pulse MIR-ON blocks. The Arduino controller generates the synchronized trigger signals for the camera and the SLM at 422.4 Hz.

In hyperspectral mode, the Arduino controller additionally controls the spectrometer and the linear translation stage of the OPO. A 19.2 Hz synchronization signal, corresponding to the volumetric acquisition rate, is sent to the spectrometer, so that each reconstructed MIP-ODT volume can be unambiguously assigned to a single MIR wavelength. The translation stage of the OPO is driven by event-triggered commands from the same timing controller.

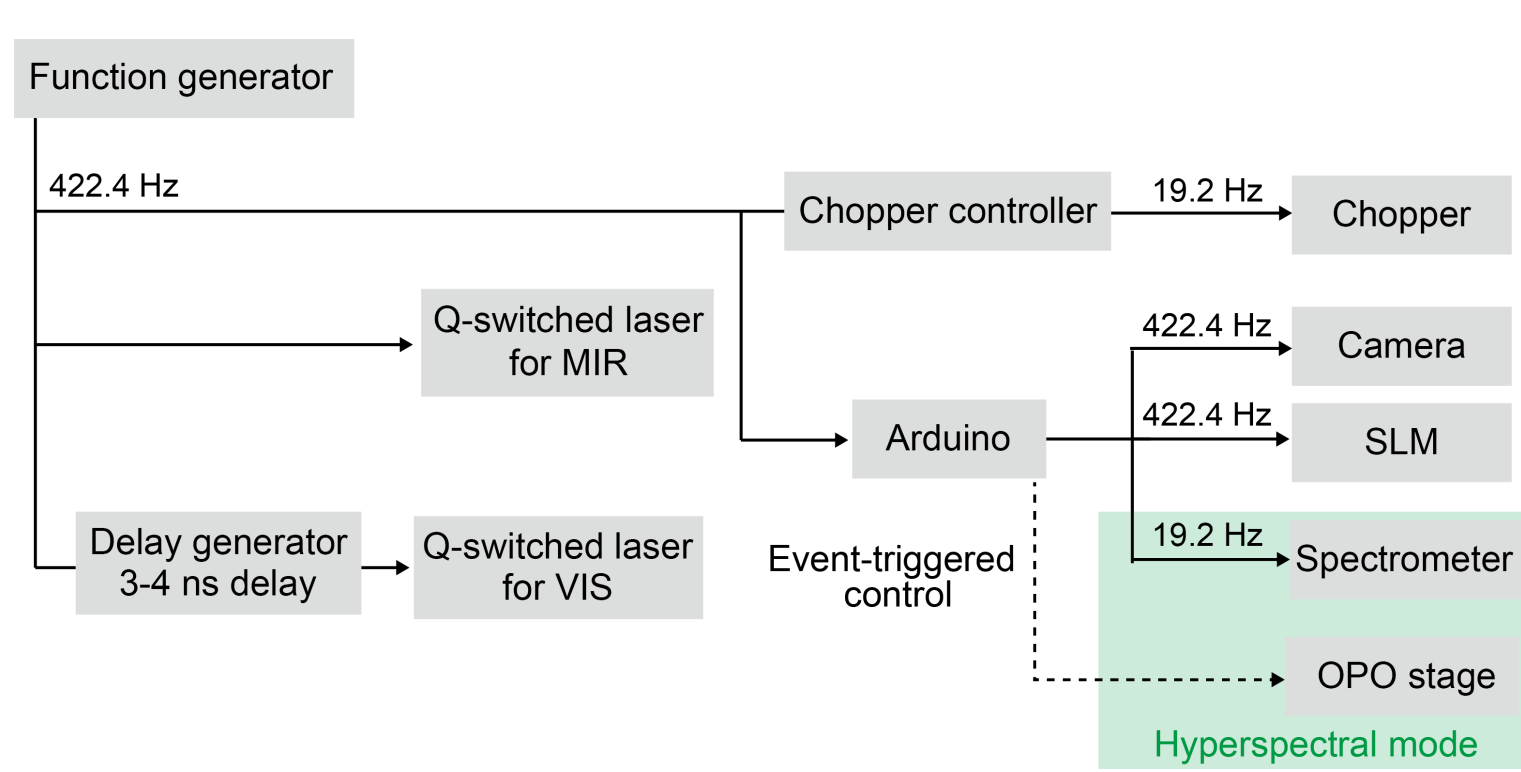

Supplementary Figure 1 | Schematic of the TTL control architecture. The 422.4 Hz master TTL clock is generated by the function generator (top-left). The solid lines represent periodic TTL signals at specified frequencies. The dashed line

represents a non-periodic, event-triggered command from the Arduino timing controller to the OPO stage. MIR, mid-infrared; SLM, spatial light modulator; VIS, visible; OPO, optical parametric oscillator.

Supplementary Note 2: Temporal stability of video-rate ODT.

We quantified the temporal RI stability of video-rate ODT. To evaluate temporal RI fluctuations, 100 volumetric datasets were acquired from a cell sample in the absence of MIR excitation at a volumetric acquisition rate of 38.4 vps. From these datasets, 50 differential RI volumes were reconstructed by subtracting each volume from its adjacent volume. The voxel-wise temporal standard deviation (STD) of these differential volumes was then calculated, providing a 3D map of ODT temporal noise. Supplementary Figure 2 shows four representative axial slices of the resulting STD map. Across most of the reconstructed volume, the STD remains below 1.0×10^{-4}. The mean STD within the entire region in Supplementary Fig. 2 is 4.2×10^{-5}. Increased noise is primarily localized at cell boundaries, where diffraction from sharp RI gradients reduces interferometric fringe visibility in the recorded holograms. The mean STD was used as the noise metric for estimating the SNR of video-rate MIP-ODT, as described in the Results section under "Validation of the spatial resolution and signal-to-noise ratio (SNR)."

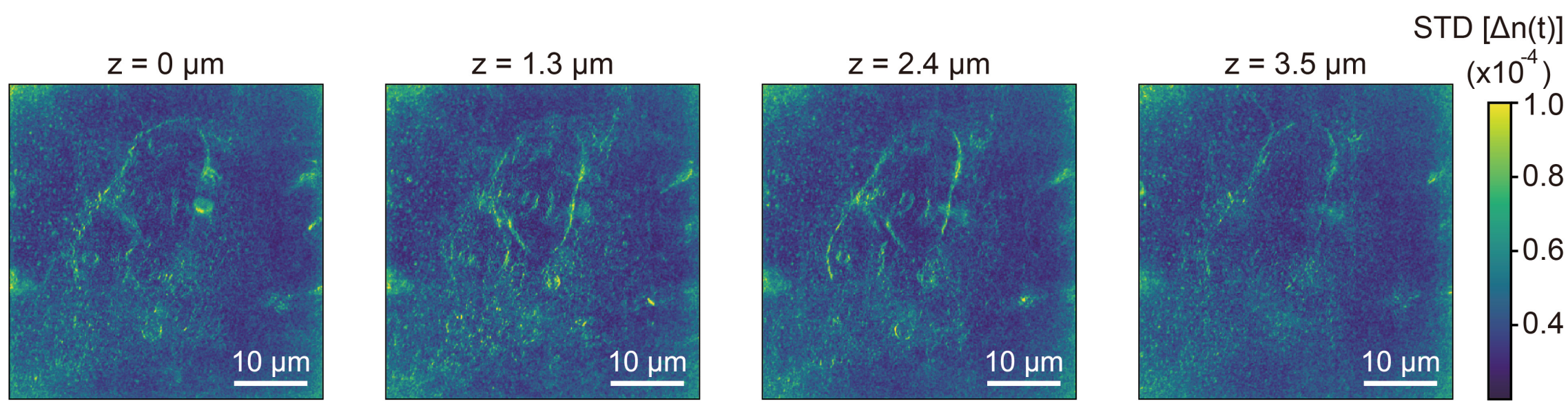

Supplementary Figure 2 | Temporal RI fluctuations in video-rate ODT. The temporal standard deviation (STD) of the differential RI signal was calculated from 50 differential RI volumes generated from 100 sequential volumetric acquisitions of a single cell sample. Scale bars, 10 μm. The axial origin is defined as the surface of the silicon substrate. The experiment was independently repeated three times with similar results.

Supplementary Note 3: High-speed MIR wavenumber sweeping.

Supplementary Figure 3 shows the MIR wavenumber trajectories inferred from the measured NIR spectra for both unidirectional fast-sweeping and continuous-sweeping modes. These measurements confirm that spectral windows of approximately 300 cm^{-1} and 200 cm^{-1} can be covered within one second under unidirectional and continuous-sweeping modes, respectively.

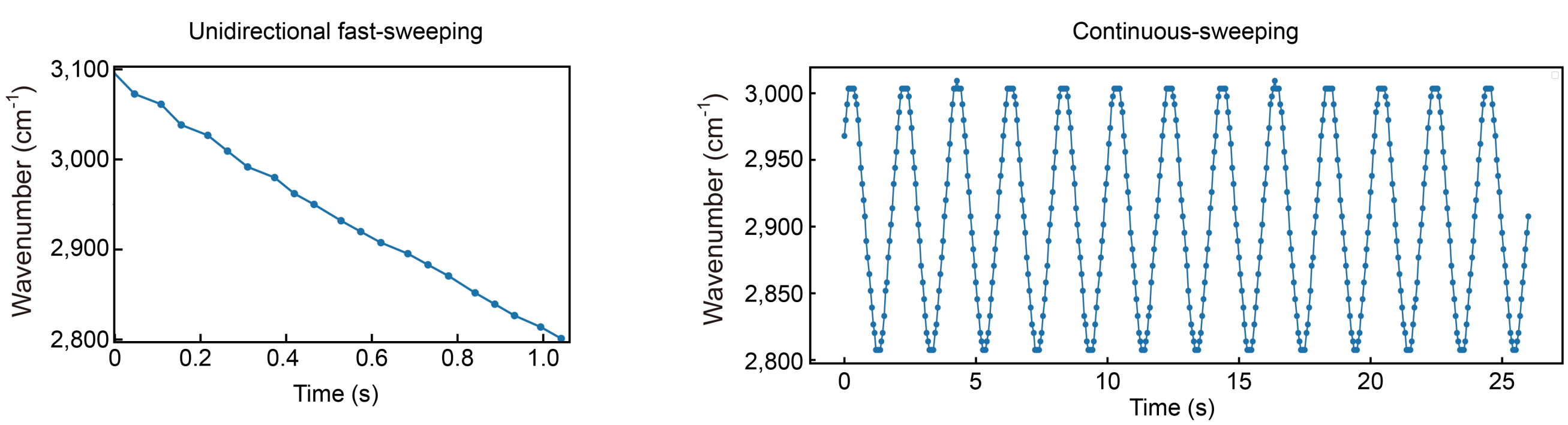

Supplementary Figure 3 | The MIR wavenumber trajectories for unidirectional fast-sweeping (left) and continuous-sweeping modes (right). Each dot was estimated from the corresponding NIR wavelength via energy conservation in the OPO. Measurements were repeated three times with similar results.

Supplementary Note 4: Direct differential reconstruction of MIP-induced RI changes.
We first summarize the general formulation of ODT. ODT is formulated as a mapping from measured complex fields to the scattering potential $F(\mathbf{r})$. Let $\mathbf{C}$ denote the set of measured complex fields. The reconstruction can be expressed as

$$F(\mathbf{r}) = \mathcal{L}(\mathbf{C}),$$

where $\mathcal{L}$ denotes the full ODT reconstruction operator. For clarity, this mapping is decomposed into two sequential steps.

First, the scattered field E^s is calculated from the measured complex field E^o and the incident field E^i, where E^i is obtained from a background measurement. We denote this step by $E^s = \mathcal{S}(E^o, E^i)$. Two standard approximations are commonly used. Under the first-order Born approximation, $E^s = E^o - E^i$, which neglects multiple scattering and is valid for weakly scattering objects. Under the first-order Rytov approximation, $E^s = \log(E^o) - \log(E^i)$, which is generally more robust for thicker samples.

Second, the retrieved scattered field is embedded into the Fourier spectrum of the scattering potential. Solving the Helmholtz equation under the weak scattering assumption yields

$$f\left(k_x - k_x^i, k_y - k_y^i, k_z\right) = -2\pi \frac{k_z}{k_m^2} e^s\left(k_x, k_y\right),$$

where $f(\mathbf{k})$ is the Fourier transform of $F(\mathbf{r})$, and $e^s(k_x, k_y)$ is the Fourier transform of the retrieved scattered field. The axial component k_z is determined by (k_x, k_y) and the incident wavevector (k_x^i, k_y^i). This embedding defines a linear operator $\mathcal{E}$ such that

$$F = \mathcal{E}(E^s). \tag{1}$$

The operator $\mathcal{E}$ includes the Fourier transformation and spectral embedding required for ODT reconstruction. Combining the two steps gives

$$F = (\mathcal{E} \circ \mathcal{S})(\mathbf{C}).$$

Next, we derive a workflow for the direct differential reconstruction of the MIP-induced RI change. The RI in the MIR-ON state is decomposed as

$$n(\mathbf{r}) = n_m + \Delta n_s(\mathbf{r}) + \Delta n_{\mathrm{MIP}}(\mathbf{r}),$$

where n_m is the RI of the surrounding medium, Δn_s denotes the static RI contrast of the sample, Δn_{MIP} represents the MIP-induced RI change. Typically, these terms satisfy $n_m \gg \Delta n_s \gg \Delta n_{\mathrm{MIP}}$. Using the scattering-potential definition,

$$F(\mathbf{r}) = \frac{k^2}{4\pi}\left(n(\mathbf{r})^2 - n_m^2\right),$$

the scattering potential in the MIR-ON state becomes

$$F_{\mathrm{ON}}(\mathbf{r}) = \frac{k^2}{4\pi}\left(\left(n_m + \Delta n_s(\mathbf{r}) + \Delta n_{\mathrm{MIP}}(\mathbf{r})\right)^2 - n_m^2\right).$$

Expanding to the first order in Δn_{MIP} yields

$$F_{\mathrm{ON}}(\mathbf{r}) = \frac{k^2}{4\pi}\left[\left(n_m + \Delta n_s(\mathbf{r})\right)^2 - n_m^2 + 2\left(n_m + \Delta n_s(\mathbf{r})\right)\Delta n_{\mathrm{MIP}}(\mathbf{r})\right] + \mathcal{O}(\Delta n_{\mathrm{MIP}}^2).$$

Neglecting the quadratic term, the scattering potential separates into static and MIP-induced components:

$$F_{\mathrm{ON}}(\mathbf{r}) \simeq F_{\mathrm{OFF}} + \Delta F_{\mathrm{MIP}},$$

where

$$\Delta F_{\mathrm{MIP}}(\mathbf{r}) = \frac{k^2}{2\pi}\left(n_m + \Delta n_s(\mathbf{r})\right)\Delta n_{\mathrm{MIP}}(\mathbf{r}).$$

We next relate ΔF_{MIP} to the measured fields using Supplementary Equation (1). Because the embedding operator $\mathcal{E}$ is linear under the weak-scattering assumption, the difference between the MIR-ON and MIR-OFF scattering potentials can be written as

$$\Delta F_{\mathrm{MIP}}(\mathbf{r}) = \mathcal{E}(E^s_{\mathrm{ON}}) - \mathcal{E}(E^s_{\mathrm{OFF}}) = \mathcal{E}(\Delta E^s_{\mathrm{MIP}}),$$

where

$$\Delta E^s_{\mathrm{MIP}} = \mathcal{S}\left(E^o_{\mathrm{ON}}, E^i\right) - \mathcal{S}\left(E^o_{\mathrm{OFF}}, E^i\right).$$

Here, E^o_{ON} and E^o_{OFF} denote the measured complex fields in the MIR-ON and MIR-OFF states, respectively. When both states share the same incident field E^i, the incident contribution cancels. Under the Born approximation, $\Delta E^s_{\mathrm{MIP}} = E^o_{\mathrm{ON}} - E^o_{\mathrm{OFF}}$, and under the Rytov approximation, $\Delta E^s_{\mathrm{MIP}} = \log\left(\frac{E^o_{\mathrm{ON}}}{E^o_{\mathrm{OFF}}}\right)$. Importantly, $\Delta E^s_{\mathrm{MIP}}$ can be calculated without explicit knowledge of E^i, enabling reconstruction based solely on the paired MIR-ON and MIR-OFF measurements. In this work, the Rytov approximation is used.

Finally, the MIP-induced RI change is obtained from

$$\Delta n_{\mathrm{MIP}}(\mathbf{r}) = \frac{2\pi}{k^2}\frac{\Delta F_{\mathrm{MIP}}}{n_m + \Delta n_s(\mathbf{r})} \simeq \frac{2\pi}{n_m k^2}\Delta F_{\mathrm{MIP}},$$

where the last approximation uses $\Delta n_s \ll n_m$.

Supplementary Note 5: Mirror-image contribution in reflection-mode ODT.

We describe the mirror-image contribution generated in reflection-mode ODT. In conventional transmission-mode ODT, the visible probe beam passes through the sample once. In contrast, in the reflection-mode ODT configuration used in this study, the visible probe beam interacts with the sample during both the incident and substrate-reflected paths (Supplementary Fig. 4a). This double-pass geometry can be unfolded into the equivalent transmission-mode representation shown in Supplementary Fig. 4a, right, in which the substrate-reflected path corresponds to propagation through a mirror-reflected object volume. Consequently, a mirror-image contribution is generated with respect to the silicon surface (Supplementary Fig. 4b). Multiple scattering effect might affect the reconstructed image quality since the ODT reconstruction algorithm relies on the weak-scattering assumption, but it is the same discussion as the conventional ODT with a thick sample.

Importantly, this contribution does not appear as an inseparable artifact in our ODT reconstruction. Supplementary Figure 4c shows a representative reconstruction of particulate objects acquired in the reflection-mode configuration. In the reconstructed 3D image, the real object and its mirror image appear at distinct axial positions. Therefore, the mirror-image contribution can be excluded by removing the corresponding axial volume from the analysis, allowing the true sample volume to be analyzed separately. However, as a caveat, because the equivalent unfolded geometry includes the mirror-reflected object volume, the effective scattering volume can be larger than in a single-pass configuration. As a result, the practical sample thickness compatible with the weak-scattering assumption may be reduced, in the same sense as conventional ODT becomes more challenging for thick or strongly scattering samples.

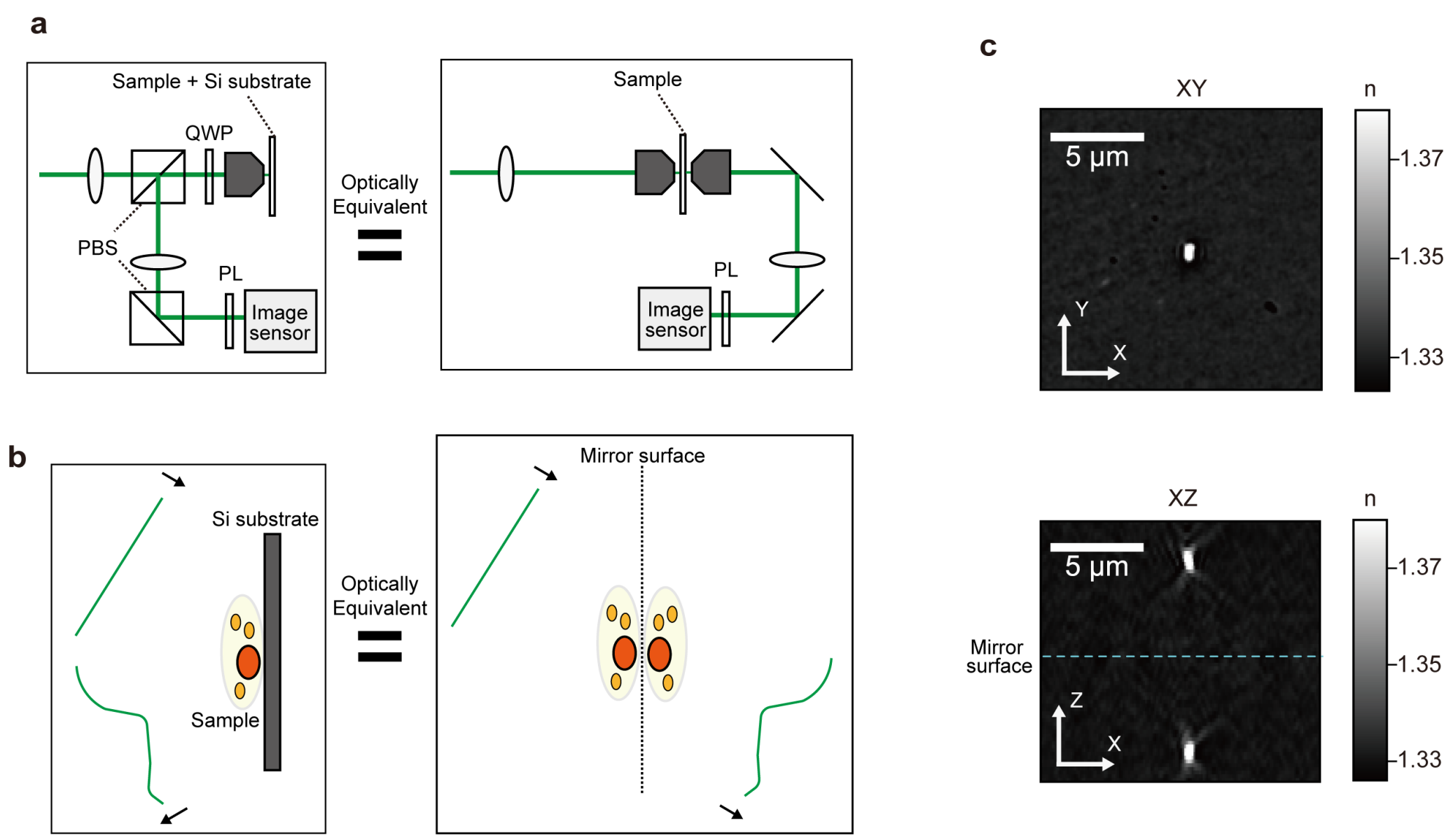

Supplementary Figure 4 | Mirror-image formation in reflection-mode ODT. a Optical configuration of the reflection-mode ODT subsystem around the sample stage. The right panel shows an equivalent transmission-mode representation. **b** Schematic representation of the corresponding sample geometry. The reconstructed object distribution is symmetric with respect to the silicon surface. **c** Planar (XY) and axial (XZ) slices of an experimentally reconstructed volume. The particulate object and its mirror image appear at symmetric axial positions relative to the reflecting surface (indicated by the blue dashed line in the XZ slice). The optical axis is parallel to the *z* direction. Scale bars, 5 µm. The grey scale represents the refractive index (n). This measurement in **c** was performed once. PBS, polarizing beam splitter; QWP, quarter-wave plate; PL, polarizer.

Supplementary Note 6: Aperture-mask design for coherent oblique illumination.

To spatially filter unwanted diffraction orders generated by the SLM, a custom aperture mask was positioned at the Fourier plane of the SLM (Supplementary Fig. 5a). The mask was fabricated from a tungsten plate with precisely machined apertures, as shown in Supplementary Fig. 5b.

Although not implemented in this study, the fixed aperture mask could be replaced by a programmable spatial filter, such as a digital micromirror device. Such an implementation would enable dynamic control of Fourier-plane filtering and scalable suppression of unwanted diffraction orders when a larger number of illumination angles is required.

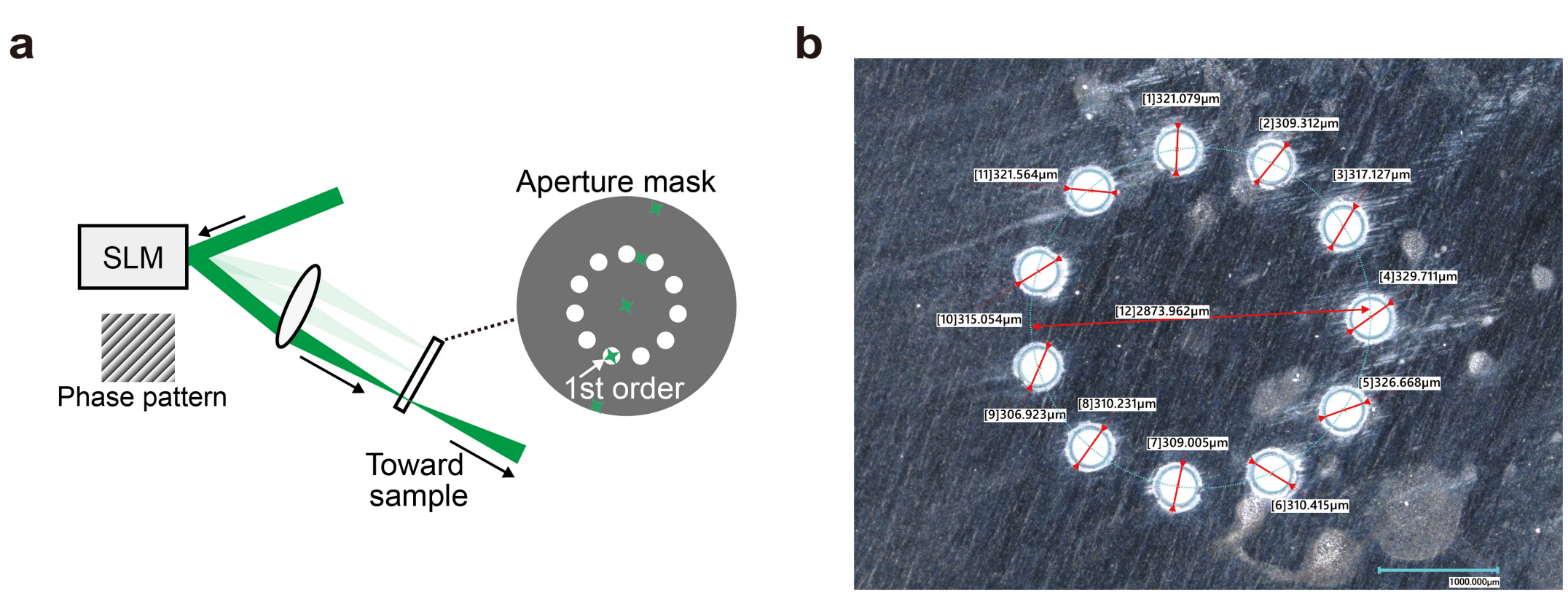

Supplementary Figure 5 | Fourier-plane aperture mask. a Optical layout showing mask placement. The green star symbols on the aperture mask represent the diffracted orders from the SLM; only the first order passes through the aperture. **b** Fabricated tungsten mask**.** Scale bar, 1 mm. The diameters of each hole and the aperture mask itself are shown in the figure. SLM, spatial light modulator.

Supplementary Note 7: Hyperspectral acquisition timing and MIR pulse-energy variation.
Supplementary Figure 6 provides a schematic timing diagram of the hyperspectral acquisition sequence and the measured MIR pulse energy as a function of wavenumber.

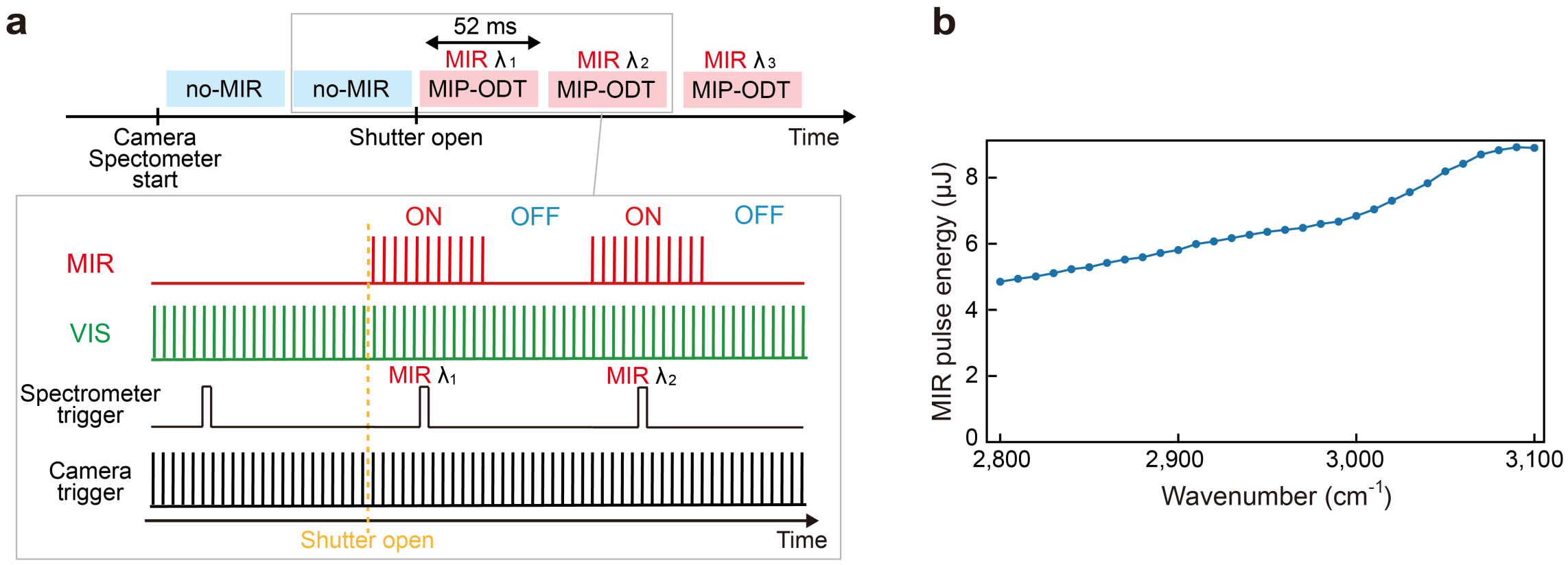

Supplementary Figure 6 | Timing of broadband multi-wavenumber spectral acquisition and MIR pulse-energy variation. a Timing diagram showing shutter opening, MIR ON/OFF acquisition, and synchronized camera and spectrometer triggers. **b** Measured MIR pulse energy as a function of wavenumber. Each data point represents a single measurement ($n = 1$ per wavenumber), obtained by dividing the average power measured with a power meter by the pulse repetition rate. MIR, mid-infrared; VIS, visible; MIP, mid-infrared photothermal; ODT, optical diffraction tomography.
