# Supplementary material for: Video-rate volumetric chemical imaging via mid-infrared photothermal optical diffraction tomography
Source: Nat Commun. 2026 Oct 30;17:11075. doi: 10.1038/s41467-026-78699-z (PMC13722699; doi:10.1038/s41467-026-78699-z)
Supplement: Supplementary file 2 [file 41467_2026_78699_MOESM2_ESM.pdf]

Description of Additional Supplementary Files

Supplementary Movie 1. MIP-ODT video with the center of mass of the analyzed particle overlaid. The color of the center-of-mass marker indicates its z position according to the color bar at the bottom. The colormap represents the MIP-induced refractive-index (RI) change.
