# Supplementary material for: Video-rate volumetric chemical imaging via mid-infrared photothermal optical diffraction tomography
Source: Nat Commun. 2026 Oct 30;17:11075. doi: 10.1038/s41467-026-78699-z (PMC13722699; doi:10.1038/s41467-026-78699-z)
Supplement: Supplementary file 4 [file 41467_2026_78699_MOESM5_ESM.pdf]

nature portfolio

Peer Review File

Video-rate volumetric chemical imaging via mid-infrared photothermal optical diffraction tomography

Corresponding Author: Professor Takuro Ideguchi

This file contains all reviewer reports in order by version, followed by all author rebuttals in order by version.

Version 0:

Reviewer comments:

Reviewer #1

(Remarks to the Author)
This paper describes a platform for rapid mid-infrared photothermal (MIP) imaging of chemicals in complex samples, such as cells. The technique provides a high signal-to-noise ratio and the capability for tomography via angular switching, and rapid wavelength scanning. The performance of the setup is validated regarding SNR, spatial resolution and hyperspectral imaging. Video-rate volumetric imaging with down to 52 ms volumetric acquisition time and tracking of lipid droplets in living cells is demonstrated.

The technical novelty of the work lies in increasing both the per frame MIP detection sensitivity by using a high-power OPO as pump laser, and the angular switching speed by employing a spatial light modulator. This is significant, because earlier setups could not perform video-rate volumetric imaging due to these limitations. Another important novelty is the introduction of a new method to determine the MIP-induced refractive index change by direct differential reconstruction, instead of subtracting separately measured MIR-OFF from MIR-ON images. The hyperspectral imaging mode allows detection of more than one chemical with one laser source, albeit at a lower image acquisition rate of about 1 s.

The significance of the work is not only that it advances the field of microspectroscopy, but also that wide-field, real-time chemical imaging and tomography as demonstrated here can lead to breakthroughs in understanding dynamic cellular phenomena.

The presented data are of high quality, technically sound and their interpretation sufficiently supports the conclusions. The analytical approach to demonstrate the MIP-ODT technique is strong and suitable. The clarity of the text and the presentation of the methods, data and results are excellent. Most question that may arise and technical issues are addressed. References and consideration of previous work are appropriate.

I strongly recommend publication of this paper in Nature Communications after considering my comments below.

1) The field-of-view was between 20x20 µm and 55x55 µm. Would an even larger field-of-view be possible with this technique and setup? What are the limiting factors?

2) In the validation of the spatial resolution, what was limiting the resolution in the axial (z) direction to around 1 µm?

3) The spectra in Fig. 3c are not well resolved, which could make interpretation difficult if two or several functional groups are present in the same volume. It would provide more confidence in the spectroscopic data if the authors would present reference spectra from pure lipids and proteins, either from the literature or measured (bulk) by conventional instruments, such as Fourier Transform Infrared (FTIR) spectroscopy.

4) Are the spectra in Fig. 3c averaged over several pixel/volumes? Was the spatial resolution in hyperspectral imaging mode the same as in the MIP-ODT validation, i.e. as stated above Fig. 2?

5) How does the direct differential reconstruction method to determine the MIP-induced refractive index change perform compared to the conventional method in terms of signal-to-noise ratio? It would be interesting to see a comparison between these two methods.

6) Since coherent probe light was used, did the authors observe any unwanted interference patterns (speckles)? Were any active measures taken to reduce the influence of speckles?

7) The platform may not be as versatile as the authors claim, as laser sources with the performance required to implement the described MIP-ODT technique are currently not available in the IR fingerprint region, where most of the cellular compounds absorb.

Reviewer #2

(Remarks to the Author)
I think this is a nice manuscript, well written and technically sound: nothing to argue about.

Regarding novelty and impact, however, I am less optimistic. The same group published a fantastic paper "Mid-infrared wide-field nanoscopy" about two years ago in Nat. Photonics (reference 19). Compared to this work, there is not much more. Yes, the authors increased the scanning rate of their ODT system to move towards video rate. This is a great technical achievement but high-speed ODT is not new. ODT+MIP is also not new, it was expertly introduced in reference 19 by the same authors.
The high speed spectral acquisition is nice, but not a shocking advancement either.

What I am missing is a convincing use case: both technical innovations are logical, published, extensions when the broader literature is consulted. Does high-speed MIP-ODT provide novel, exciting or ground-breaking insight? Looking at the data provided I, unfortunately, was not able to identify convincing demonstrations. The authors track a few lipid droplets that appeared near-stationary within the depth of field of the imaging system and did not even perform in-depth analysis of their 3D motion: why track in 3D in the first place? 2D observations would have yielded >200 FPS and the same information content. Why not track over minutes to showcase true 3D capabilities?
The same goes for the spectral aspects: it is fast but no dynamics are presented, so what is the benefit?

At its current stage, I believe that this is a fantastic technical contribution with a lot of potential. For a broad-audience journal, however, the broad-audience reader needs to see results that convincingly showcase the capabilities in an exciting manner that goes beyond the x-times faster acquisition. My feeling is that a lot of painful assay-work would be necessary to provide this insight.

Reviewer #3

(Remarks to the Author)
The work explores volumetric spectral imaging by combining a photothermal approach with diffraction optical tomography. The authors experimentally demonstrate that reducing the number of acquisition angles can provide a practical compromise between image quality and imaging speed. While the results are interesting and promising, the manuscript would benefit significantly from improvements in both presentation and technical clarity.

Major Comments:

1. Positioning and scope of the technique. The work is presented as video-rate volumetric imaging, drawing parallels to the field of mid-infrared optical coherence tomography (Optical Coherence Tomography). This framing is potentially misleading. The demonstrated imaging volumes are limited to samples on the order of ~10 um in thickness, essentially at the scale of a single cell layer. While the method successfully captures lipid droplet dynamics within this confined volume, this represents a highly specific use case. It remains unclear whether the approach can be extended to thicker samples or true tissue-scale imaging, which is a defining strength and goal of OCT-based methodologies. The limitations imposed by photothermal contrast, particularly with respect to sample thickness and background contributions are well known but not sufficiently addressed here. At minimum, the manuscript (including the title) should be revised to more accurately reflect the demonstrated regime and avoid overgeneralization.

2. Imaging contrast and data interpretation. This is a central concern:
a. The images are difficult to interpret, particularly in comparative contexts (e.g., Figure 3b). The current visualization choices obscure rather than clarify the results. For example, the color palette is poorly selected: the 2932 cm-1 resonance is shown using a two-color scheme, while the 2962 cm-1 signal is rendered in a single green tone. The rationale for this distinction is unclear, and it is difficult to assess whether the observed differences are physically meaningful.
b. Very important, the presented data suggest a significant background contribution. To properly evaluate chemical contrast, it would be essential to include side-by-side comparisons of CH_2 and CH_3 resonances, along with an off-resonance condition (e.g., near 3000 cm-1, as partially suggested in Figure 3c). The doemsntration of signal-to-background is of particular importance.
c. The CH_3 stretching mode appears substantially suppressed relative to the CH_2 mode (~2932 cm^{-1}). This is unexpected and should be explained or supported with additional data.
d. The imaging geometry introduces further complexity. The visible probe beam traverses the sample twice at an angle (incoming and reflected from silicon substrate), complicating interpretation of spatial localization, lateral and axial resolution, and spectral response, particularly in angle-dependent reconstructions. This geometry may introduce additional background or “haze,” which could explain some of the observed image features. A more rigorous treatment or discussion is necessary.

3. System characterization. While the system is characterized, it is not benchmarked against standard or quasi-standard samples. For a newly proposed imaging modality, it is important to establish spatial resolution and contrast performance using well-defined structures. The current demonstrations shows resolution of single lipid droplet, with surrounding media not interfering with the signals (neight scattering nor thermal management, an important parameter for photothermal imaging). As a result, it is difficult to disentangle true resolution limits from sample-dependent effects. Step-function targets or sharp interfaces between two bulks for spatial resolution would be particularly valuable here.

4. Signal metrics. The manuscript emphasizes signal-to-noise ratio as one of a key performance metric. However, for chemically selective imaging, signal-to-background ratio is often more relevant. Demonstrating strong chemical contrast requires clear suppression of background signals, which is not convincingly shown in the current data.

Minor Comments:

1. Terminology and claims. The term “real-time hyperspectral imaging” is not used accurately. First, the method employs discrete wavelengths and is therefore more appropriately described as spectral rather than hyperspectral imaging. Second, the reported acquisition time (~1 second per spectral stack) does not meet the conventional definition of real-time or video-rate spectral imaging (>20 frames per second).

2. Several figures include elements that are not adequately described in the text or captions. For example:
a. Figure 3c: the acquisition time point is not specified.
b. Figure 4a: the meaning of the dotted circle is unclear.

3. The discussion of photothermal imaging omits a foundational reference. While many follow-up works are cited, the original demonstration of the technique should be included -https://doi.org/10.1364/OE.19.001378

4.The manuscript does not adequately represent the current state of the field. For example, CARS is not the most recent advancement in this space. More recent developments should be acknowledged, including:
a. Upconversion imaging using external media - https://doi.org/10.1364/JOSAA.393580
b. Non-degenerate two-photon absorption -based imaging, which enables volumetric and spectral modalities using standard semiconductor detectors - https://doi.org/10.1364/OPTICA.426199 and https://www.science.org/doi/10.1126/sciadv.ade4247

Version 1:

Reviewer comments:

Reviewer #1

(Remarks to the Author)
The authors have satisfactorily answered the reviewers' comments and carefully revised the manuscript. I recommend publication.

Reviewer #2

(Remarks to the Author)

Reviewer #3

(Remarks to the Author)
The authors have addressed most of my comments, and while a few questions remain, I do not believe they affect the robustness of the study or the validity of the results presented. Overall, I find this to be a solid and well-executed piece of work.
My only remaining concern is whether the manuscript is a sufficiently strong fit for Nature Communications. Similar to Reviewer 2, I remain somewhat concerned about the level of novelty expected for this journal. In my view, the present study represents primarily a logical and relatively routine extension of the authors’ previous work rather than a sufficiently substantial conceptual advance.

Open Access This Peer Review File is licensed under a Creative Commons Attribution 4.0 International License, which permits use, sharing, adaptation, distribution and reproduction in any medium or format, as long as you give appropriate credit to the original author(s) and the source, provide a link to the Creative Commons license, and indicate if changes were made.
In cases where reviewers are anonymous, credit should be given to 'Anonymous Referee' and the source.
The images or other third party material in this Peer Review File are included in the article's Creative Commons license, unless indicated otherwise in a credit line to the material. If material is not included in the article's Creative Commons license and your intended use is not permitted by statutory regulation or exceeds the permitted use, you will need to obtain permission directly from the copyright holder.
To view a copy of this license, visit https://creativecommons.org/licenses/by/4.0/

To Reviewer 1

We are grateful to the Reviewer for taking the time to review our manuscript and give us his/her valuable comments. Our point-by-point responses to the Reviewer's comments are shown below.

Reviewer 1's comment #1:

This paper describes a platform for rapid mid-infrared photothermal (MIP) imaging of chemicals in complex samples, such as cells. The technique provides a high signal-to-noise ratio and the capability for tomography via angular switching, and rapid wavelength scanning. The performance of the setup is validated regarding SNR, spatial resolution and hyperspectral imaging. Video-rate volumetric imaging with down to 52 ms volumetric acquisition time and tracking of lipid droplets in living cells is demonstrated.

The technical novelty of the work lies in increasing both the per frame MIP detection sensitivity by using a high-power OPO as pump laser, and the angular switching speed by employing a spatial light modulator. This is significant, because earlier setups could not perform video-rate volumetric imaging due to these limitations. Another important novelty is the introduction of a new method to determine the MIP-induced refractive index change by direct differential reconstruction, instead of subtracting separately measured MIR-OFF from MIR-ON images. The hyperspectral imaging mode allows detection of more than one chemical with one laser source, albeit at a lower image acquisition rate of about 1 s.

The significance of the work is not only that it advances the field of microspectroscopy, but also that wide-field, real-time chemical imaging and tomography as demonstrated here can lead to breakthroughs in understanding dynamic cellular phenomena.

The presented data are of high quality, technically sound and their interpretation sufficiently supports the conclusions. The analytical approach to demonstrate the MIP-ODT technique is strong and suitable. The clarity of the text and the presentation of the methods, data and results are excellent. Most question that may arise and technical issues are addressed. References and consideration of previous work are appropriate.

I strongly recommend publication of this paper in Nature Communications after considering my comments below.

Authors' response:

We sincerely thank the reviewer for the positive assessment of our work and for recommending publication. We have carefully considered the reviewer's comments and respond to each point below.

Reviewer 1's comment #2:

1) *The field-of-view was between 20x20 µm and 55x55 µm. Would an even larger field-of-view be possible with this technique and setup? What are the limiting factors?*

Authors' response:

We thank the reviewer for raising this important point. The field of view can be further expanded using the present technique and experimental configuration. In practice, the achievable field of view is determined primarily by the available MIR pulse energy and the MIP signal-to-noise ratio required for reliable imaging. At a fixed MIR pulse energy, increasing the illuminated area reduces the MIR fluence, which in turn decreases the photothermally induced refractive-index change and the detected MIP signal.

For the video-rate measurements in Fig. 2, we achieved an SNR of approximately 74 over a field of view of approximately 20 × 20 μm^2. Assuming uniform illumination and unchanged detection noise, this SNR margin indicates that the current MIR source could support a field of view of approximately 55 × 55 μm^2 while maintaining an SNR of approximately 10. A still larger field of view should be achievable using higher-pulse-energy MIR sources, as tunable fan-out MgO:PPLN OPOs delivering several hundred microjoules per pulse have been demonstrated [Ref. 1].

The field of view reported in this study therefore does not represent an intrinsic limit of MIP-ODT. Rather, it reflects the balance among the available MIR pulse energy, acquisition speed, illuminated area, and required MIP sensitivity under the imaging conditions used here.

[Ref. 1] Xiong, B. *et al.* High-power, high-repetition-rate operation of a period-continuously-tunable optical parametric oscillator at 3.78–4.62 μm based on a fan-out periodically poled MgO-doped lithium niobate. *Laser Phys.* **21**, 362–366 (2011).

Reviewer 1's comment #3:

2) In the validation of the spatial resolution, what was limiting the resolution in the axial (z) direction to around 1 μm?

Authors' response:

We thank the reviewer for this important question. The axial resolution of approximately 1 μm in MIP-ODT was limited by the spatial resolution of the underlying ODT reconstruction, rather than by the MIP contrast mechanism itself. In ODT, this resolution is governed by the probe wavelength, the collection NA, and the angular range of illumination, which together define the accessible region of the object spectrum in 3D Fourier space.

In the present experiment, the main factor limiting the ODT resolution was the modest illumination NA. We chose this illumination pattern to balance axial resolution, lateral field of view, and observable depth range for single-cell volumetric imaging. Increasing the illumination NA would improve axial frequency coverage and thereby enhance axial resolution. However, high-angle illumination also narrows the practical volume over which the scattered field can be efficiently collected and reconstructed, resulting in a tradeoff with the lateral field of view and observable depth.

The present system has not yet reached the ultimate resolution achievable with this optical configuration. Numerical evaluation of the point-spread function calculated from the 3D Fourier-space coverage indicates that, by increasing the maximum illumination NA from 0.85 to 1.2 while keeping the current optical setup, the theoretical axial resolution would improve to approximately 300–400 nm. Thus, the approximately 1-µm axial resolution reported in this work reflects the illumination condition selected to maintain a practical balance among axial resolution, lateral field of view, and observable depth for single-cell volumetric imaging, rather than a fundamental resolution limit of MIP-ODT. We have clarified this point in the Discussion section of the revised manuscript.

Reviewer 1's comment #4:

3) *The spectra in Fig. 3c are not well resolved, which could make interpretation difficult if two or several functional groups are present in the same volume. It would provide more confidence in the spectroscopic data if the authors would present reference spectra from pure lipids and proteins, either from the literature or measured (bulk) by conventional instruments, such as Fourier Transform Infrared (FTIR) spectroscopy.*

Authors' response:

We thank the reviewer for this important suggestion. We agree that reference spectra are valuable for interpreting partially overlapping vibrational bands and for assessing the reliability of local spectroscopic assignments. In the revised manuscript, we have therefore added reference spectra of representative protein-, nucleic-acid-, and lipid-related samples to Fig. 3.

We also used these spectra as references for non-negative least-squares (NNLS) fitting of the local MIP spectra. For the nucleolus, we used bovine serum albumin, guanosine, and oleic acid as representative references for protein-like, nucleic-acid-like, and lipid-like spectral contributions, respectively. The fitted spectrum closely reproduced the measured nucleolar spectrum, with only a small residual. The normalized fitting coefficients indicated a dominant protein-like contribution, a smaller nucleic-acid-related contribution, and only a minor lipid-like contribution. This result is consistent with the protein- and RNA-rich composition expected for the nucleolus.

For the small cytoplasmic particle assigned as a lipid droplet, we performed two-component NNLS fitting using oleic acid and cholesterol as representative references for unsaturated acyl-chain-like and sterol-like spectral contributions, respectively. The fitted spectrum closely reproduced the measured local spectrum and allowed us to distinguish the relative contributions associated with the two reference components, providing a more detailed spectral characterization of the lipid droplet.

Reviewer 1's comment #5:

4) *Are the spectra in Fig. 3c averaged over several pixel/volumes? Was the spatial resolution in hyperspectral imaging mode the same as in the MIP-ODT validation, i.e. as stated above Fig. 2?*

Authors' response:

We thank the reviewer for this question. Yes, the spectra shown in Fig. 3c were averaged over selected regions of interest. We have revised the figure caption to specify the number of pixels/voxels averaged for each spectrum.

Regarding the spatial resolution in the hyperspectral imaging mode, the same optical configuration and illumination pattern as those used for the MIP-ODT resolution validation in Fig. 2 were employed. Therefore, the spatial resolution in the hyperspectral measurements was the same as that reported in Fig. 2.

Reviewer 1's comment #6:

5) *How does the direct differential reconstruction method to determine the MIP-induced refractive index change perform compared to the conventional method in terms of signal-to-noise ratio? It would be interesting to see a comparison between these two methods.*

Authors' response:

We thank the reviewer for this question. As derived in Supplementary Note 4, direct differential reconstruction is based on the exchangeability of linear operations. Under ideal conditions, it gives the same intrinsic signal-to-noise ratio as the conventional reconstruction-then-subtraction approach. However, in experimental MIP-ODT, the MIR-ON and MIR-OFF phase images can contain small phase offsets and slowly varying phase gradients that are unrelated to the photothermal signal. These residual phase errors mainly arise from interferometer fluctuations caused by mechanical instability and air-current disturbances. Because the MIP-induced refractive-index change is small, these residual phase errors can become comparable to or larger than the true MIP signal. In the conventional reconstruction-then-subtraction approach, such errors can propagate through the two separate 3D reconstructions and appear as non-photothermal artifacts in the final subtracted volume.

Direct differential reconstruction is therefore essential in practice because it allows these offset and gradient errors to be corrected before 3D reconstruction, preventing such artifacts from propagating into the reconstructed MIP volume. We have clarified this practical necessity of direct differential reconstruction in the revised manuscript.

Reviewer 1's comment #7:

6) *Since coherent probe light was used, did the authors observe any unwanted interference patterns (speckles)? Were any active measures taken to reduce the influence of speckles?*

Authors' response:

We thank the reviewer for this question. Because coherent probe light was used, we initially observed unwanted interferometric noise, mainly arising from multiple reflections in the optical system. To reduce this effect, we introduced a 10-m single-mode fiber between the light source and the interferometer. Nonlinear optical effects in the

fiber broadened the spectral bandwidth of the probe light, thereby shortening the coherence length. As a result, interference signals caused by unwanted multiple reflections were substantially suppressed.

At the same time, an excessively short coherence length would reduce the visibility of the intended off-axis interferometric signal. Therefore, after spectral broadening in the fiber, we adjusted the probe bandwidth using a 2-nm full-width-at-half-maximum bandpass filter. This configuration shortened the coherence length sufficiently to suppress unwanted interference from multiple reflections, while preserving adequate temporal coherence for stable off-axis digital holographic detection.

Reviewer 1's comment #8:

7) The platform may not be as versatile as the authors claim, as laser sources with the performance required to implement the described MIP-ODT technique are currently not available in the IR fingerprint region, where most of the cellular compounds absorb.

Authors' response:

We thank the reviewer for raising this important point. We agree that the accessible spectral range of the present implementation is determined by the MIR laser source. In this study, we employed a nanosecond OPO based on a fan-out PPLN crystal, and therefore the MIR output was limited to the high-wavenumber region.

However, this limitation originates from the specific nonlinear crystal used in the present OPO, rather than from the MIP-ODT platform itself. The nanosecond pulse duration, kilohertz repetition rate, and microjoule-level pulse energy required for the present MIP-ODT measurement are not fundamentally limited to this spectral region. Indeed, nanosecond MIR−OPO sources based on nonlinear crystals such as AGS and BGSe have been experimentally demonstrated for longer MIR wavelengths, including the fingerprint region [Refs. 2, 3].

Therefore, fingerprint-region MIP-ODT should be feasible by replacing the PPLN-based OPO with an appropriate fingerprint-region nanosecond MIR source. We have revised the manuscript to clarify that the spectral range demonstrated in this work is limited by the current light source, and that extension to the fingerprint region requires an MIR source optimized for that spectral range.

[Ref. 2] Vodopyanov, K. L. *et al.* $AgGaS_2$ optical parametric oscillator continuously tunable from 3.9 to 11.3 μm. *Appl. Phys. Lett.* **75**, 1204–1206 (1999).

[Ref. 3] Petrov, V. *et al*. Barium nonlinear optical crystals for the mid-IR: characterization and some applications. *J. Opt. Soc. Am. B* **38**, B46–B58 (2021).

To Reviewer 2

We are grateful to the Reviewer for taking the time to review our manuscript and give us his/her valuable comments. Our point-by-point responses to the Reviewer's comments are shown below.

Reviewer 2's comment #1:

I think this is a nice manuscript, well written and technically sound: nothing to argue about.

Regarding novelty and impact, however, I am less optimistic. The same group published a fantastic paper "Mid-infrared wide-field nanoscopy" about two years ago in Nat. Photonics (reference 19). Compared to this work, there is not much more. Yes, the authors increased the scanning rate of their ODT system to move towards video rate. This is a great technical achievement but high-speed ODT is not new. ODT+MIP is also not new, it was expertly introduced in reference 19 by the same authors.

The high speed spectral acquisition is nice, but not a shocking advancement either.

Authors' response:

We thank the reviewer for the positive assessment of our manuscript and for raising this important question regarding its novelty and impact. We respectfully clarify that the main advance of the present work is not simply a higher angular scanning rate of the visible probe beam. First of all, Ref. 19 did not demonstrate MIP-ODT. In that study, multi-angle illumination was used for synthetic-aperture QPI to improve the lateral resolution of two-dimensional MIP images. By contrast, the present method reconstructs the three-dimensional distribution of the MIP-induced refractive-index change using ODT.

We agree that related techniques, including synthetic-aperture MIP-QPI [Ref. 19] and high-speed ODT, have been reported previously. However, combining their individual capabilities does not directly yield high-speed MIP-ODT. The central challenge is to detect weak MIP-induced phase changes with high phase stability while rapidly changing the illumination angle. For each illumination angle, an angle-resolved frame must be acquired using a single short visible probe pulse synchronized with the MIR excitation, while preserving a sufficient photon budget for sensitive phase detection. The acquisition sequence must also be synchronized with the AC drive cycle of the spatial light modulator to suppress frame-to-frame phase fluctuations. In addition, time-varying coherent artifacts caused by multiple reflections must be minimized because these artifacts directly contaminate the MIR-ON/MIR-OFF differential phase measurements. The resulting weak, angle-resolved differential phase signals must then be quantitatively converted into a three-dimensional distribution of the MIP-induced refractive-index change using a dedicated differential tomographic reconstruction framework.

These constraints are specific to high-speed MIP tomography and are not generally encountered in conventional high-speed ODT. High-speed MIP-ODT therefore cannot be realized by simply adding MIR excitation to existing high-speed ODT systems. Similarly, merely increasing the angular scanning speed of the synthetic-aperture MIP-QPI approach used in Ref. 19 would not be sufficient. The present study addresses this technical gap by integrating phase-stable, high-speed, angle-resolved MIP acquisition with quantitative differential reconstruction, thereby enabling

video-rate volumetric chemical imaging. We have clarified these distinctions and the resulting technical advance in the Discussion and Methods sections of the revised manuscript.

Reviewer 2's comment #2:
What I am missing is a convincing use case: both technical innovations are logical, published, extensions when the broader literature is consulted. Does high-speed MIP-ODT provide novel, exciting or ground-breaking insight? Looking at the data provided I, unfortunately, was not able to identify convincing demonstrations. The authors track a few lipid droplets that appeared near-stationary within the depth of field of the imaging system and did not even perform in-depth analysis of their 3D motion: why track in 3D in the first place? 2D observations would have yielded >200 FPS and the same information content. Why not track over minutes to showcase true 3D capabilities?

The same goes for the spectral aspects: it is fast but no dynamics are presented, so what is the benefit?

Authors' response:
We thank the reviewer for this important comment. We respectfully clarify that the lipid-droplet tracking experiment in Fig. 4 was intended to demonstrate a biologically relevant use case of video-rate MIP-ODT. Lipid droplets are particularly suitable targets for label-free vibrational imaging because their spatial distribution, intracellular transport, and structural remodeling are closely linked to cellular metabolism and to interactions with organelles, the cytoskeleton, and local intracellular environments. Video-rate MIP-ODT allows lipid droplets to be visualized through their intrinsic chemical contrast and tracked in three dimensions without exogenous labels. We have revised the corresponding text in the manuscript to clarify the motivation and biological relevance of this experiment.

The main significance of the present experiment lies not in the magnitude of the observed displacement itself, but in the quantitative three-dimensional characterization of the transport behavior of chemically identified lipid droplets. Even within the short acquisition window, the tracked droplets showed subdiffusive motion and heterogeneous intracellular mobility. Because lipid-droplet mobility is influenced by interactions with the cytoskeleton, organelles, and the surrounding cytoplasm, these transport parameters provide information about the local intracellular environment encountered by individual droplets. Three-dimensional tracking also resolves directional differences in mobility that would be obscured in a two-dimensional projection.

To highlight the additional information provided by three-dimensional tracking, we have added two complementary analyses of a representative lipid-droplet trajectory to the revised manuscript. First, the trajectory exhibited local fluctuations around transient centroid positions together with a step-like relocation between them. The centroid shift was approximately 0.28 μm in three dimensions and could be fully quantified only by measuring the particle position along all three spatial axes. Second, we calculated the mean-squared displacement separately along each Cartesian axis to assess the directional dependence of the motion. The anomalous exponents differed markedly among the three axes, with $\alpha_X = 0.68$, $\alpha_Y = 0.34$, and $\alpha_Z = 0.14$, indicating strongly anisotropic transport. Three-dimensional MIP

imaging can resolve step-like relocations and directional heterogeneity in lipid-droplet transport, both of which may be underestimated or missed in two-dimensional observations.

Finally, we agree that minute-scale tracking and dynamic multi-wavenumber spectral imaging would provide a stronger biological demonstration of the platform. In the present setup, however, continuous acquisition was limited to approximately 3 s because the commercial SLM-based beam-steering system used in this study could not reliably update its phase patterns during sustained high-speed operation. This limitation arose from a system-specific technical issue involving the device and its control implementation and was neither operator-dependent nor an inherent limitation of MIP-ODT. Although it should be addressable through improved SLM control or alternative high-speed beam-steering hardware, it could not be resolved immediately within the scope of the present study. We have therefore expanded the Discussion to identify minute-scale three-dimensional tracking and dynamic multi-wavenumber monitoring as important directions for future development.

Reviewer 2's comment #3:

At its current stage, I believe that this is a fantastic technical contribution with a lot of potential. For a broad-audience journal, however, the broad-audience reader needs to see results that convincingly showcase the capabilities in an exciting manner that goes beyond the x-times faster acquisition. My feeling is that a lot of painful assay-work would be necessary to provide this insight.

Authors' response:

We thank the reviewer for this constructive comment. We agree that a broad-audience journal requires a compelling use case beyond faster acquisition alone. As discussed in our response to Comment 2, the lipid-droplet tracking experiment demonstrates such a use case by enabling label-free 3D chemical tracking of intracellular lipid-rich structures and quantification of their diffusion behavior. We also note that the absence of longer-term tracking in the present study reflects an implementation-specific limitation of the current commercial SLM, rather than a fundamental limitation of the MIP-ODT principle. We have revised the manuscript to better clarify the biological motivation and quantitative value of the lipid-droplet tracking experiment.

To Reviewer 3

We are grateful to the Reviewer for taking the time to review our manuscript and give us his/her valuable comments. Our point-by-point responses to the Reviewer's comments are shown below.

Reviewer 3's comment #1:

The work explores volumetric spectral imaging by combining a photothermal approach with diffraction optical tomography. The authors experimentally demonstrate that reducing the number of acquisition angles can provide a practical compromise between image quality and imaging speed. While the results are interesting and promising, the manuscript would benefit significantly from improvements in both presentation and technical clarity.

Authors' response:

We thank the reviewer for the positive assessment and constructive comments. In the revised manuscript, we have improved the presentation and technical clarity by revising the descriptions of the imaging geometry, reconstruction procedure, system characterization, signal metrics, terminology, and figure captions.

Reviewer 3's comment #2:

1. Positioning and scope of the technique. The work is presented as video-rate volumetric imaging, drawing parallels to the field of mid-infrared optical coherence tomography (Optical Coherence Tomography). This framing is potentially misleading. The demonstrated imaging volumes are limited to samples on the order of ~10 um in thickness, essentially at the scale of a single cell layer. While the method successfully captures lipid droplet dynamics within this confined volume, this represents a highly specific use case. It remains unclear whether the approach can be extended to thicker samples or true tissue-scale imaging, which is a defining strength and goal of OCT-based methodologies. The limitations imposed by photothermal contrast, particularly with respect to sample thickness and background contributions are well known but not sufficiently addressed here. At minimum, the manuscript (including the title) should be revised to more accurately reflect the demonstrated regime and avoid overgeneralization.

Authors' response:

We thank the reviewer for raising this point. We first clarify that ODT in the present manuscript refers to optical diffraction tomography, not optical coherence tomography. The manuscript does not draw a parallel between MIP-ODT and mid-infrared OCT or position our method as a platform for deep tissue imaging. These two tomographic approaches operate in distinct imaging regimes. OCT is primarily used for depth-resolved imaging in optically scattering tissues, whereas ODT is a microscopy technique that reconstructs three-dimensional refractive-index distributions in microscopic specimens, including single cells and thin biological samples. The objective of the present work is therefore high-resolution volumetric chemical imaging at the single-cell scale, rather than tissue-scale imaging.

We agree, however, that the applicable sample-thickness range and the meaning of "volumetric imaging" should be stated more explicitly. In this study, the term refers to the reconstruction of three-dimensional chemical contrast within a microscopic sample volume and does not imply deep tissue penetration. This usage is consistent with its

established meaning in optical microscopy, where volumetric imaging includes three-dimensional imaging of single cells and other microscopic specimens. We have therefore retained the term "volumetric" in the title while revising the manuscript to define the demonstrated regime more clearly as single-cell-scale volumetric MIP imaging, demonstrated here for sample thicknesses ranging from several micrometers to approximately 10 µm, with potential applicability to specimens several tens of micrometers thick.

We have also expanded the Discussion to describe the factors that limit extension to substantially thicker specimens. These include attenuation of the MIR excitation, increased background photothermal signals, scattering of the visible probe light, and eventual breakdown of the weak-scattering approximation used for tomographic reconstruction. These revisions more clearly define the scope of the present method and distinguish it from tissue-scale tomographic imaging.

Reviewer 3's comment #3:

2. Imaging contrast and data interpretation. This is a central concern:

a. The images are difficult to interpret, particularly in comparative contexts (e.g., Figure 3b). The current visualization choices obscure rather than clarify the results. For example, the color palette is poorly selected: the 2932 cm-1 resonance is shown using a two-color scheme, while the 2962 cm-1 signal is rendered in a single green tone. The rationale for this distinction is unclear, and it is difficult to assess whether the observed differences are physically meaningful.

Authors' response:

We agree that the previous visualization made it difficult to compare the CH_2 and CH_3 images directly. We have therefore revised the color mapping so that the CH_2 and CH_3 images are displayed using the same colormap. This provides a more consistent visual representation and enables clearer side-by-side comparison of the chemical contrast.

Reviewer 3's comment #4:

b. Very important, the presented data suggest a significant background contribution. To properly evaluate chemical contrast, it would be essential to include side-by-side comparisons of CH2 and CH3 resonances, along with an off-resonance condition (e.g., near 3000 cm-1, as partially suggested in Figure 3c). The doemsntration of signal-to-background is of particular importance.

Authors' response:

We agree that evaluating the background contribution is essential for assessing chemical contrast. In the revised manuscript, we have added side-by-side comparisons of the images acquired at 2,932 cm^{-1}, 2,962 cm^{-1}, and the off-resonance condition at 3,106 cm^{-1}. These comparisons show that the resonant MIP signals are clearly distinguishable from the off-resonance background. We also quantified the signal-to-background ratio from a cytoplasmic particle relative to the spatially averaged background in the off-resonant 3,106 cm^{-1} image, yielding an SBR of 56 (see Comment 8).

Reviewer 3's comment #5:

c. The CH3 stretching mode appears substantially suppressed relative to the CH2 mode (~2932 cm^{-1}). This is unexpected and should be explained or supported with additional data.

Authors' response:

We thank the reviewer for this important comment. We understand the reviewer's concern as referring to the weaker signal near 2,962 cm^{-1} relative to the CH_2-associated band near 2,932 cm^{-1} in the small cytoplasmic particle. We agree that this spectral contrast should be clarified.

A relatively weak signal near 2,962 cm^{-1} is expected for lipid-rich compartments such as lipid droplets. Lipid droplets are primarily composed of neutral lipids containing long fatty-acyl chains, in which methylene groups are abundant along the hydrocarbon chains, whereas methyl groups are largely confined to the chain termini. As a result, lipid-rich materials typically show strong CH_2 stretching bands near 2,850 and 2,920–2,930 cm^{-1}, while the CH_3-associated contribution near 2,950–2,960 cm^{-1} is comparatively weaker. This spectral tendency is also supported by the oleic acid reference spectrum added to the revised manuscript. We have revised the manuscript to clarify this spectral interpretation and to support it with the added oleic acid reference spectrum.

Reviewer 3's comment #6:

d. The imaging geometry introduces further complexity. The visible probe beam traverses the sample twice at an angle (incoming and reflected from silicon substrate), complicating interpretation of spatial localization, lateral and axial resolution, and spectral response, particularly in angle-dependent reconstructions. This geometry may introduce additional background or "haze," which could explain some of the observed image features. A more rigorous treatment or discussion is necessary.

Authors' response:

We thank the reviewer for raising this important point. We agree that the reflection-mode geometry introduces additional complexity because the visible probe beam interacts with the sample during both the incident and substrate-reflected paths. This geometry can generate a mirror-image contribution relative to the silicon substrate, as illustrated in the figure below.

Importantly, this contribution does not appear as an unresolved haze in our reconstruction. In the reconstructed 3D volume, the true object and its mirror image are separated along the axial direction. As shown below, the mirror image appears at an axial position distinct from the actual sample volume, indicating that the two contributions can be spatially resolved and distinguished in 3D. We have clarified this point in the Methods section of the revised manuscript.

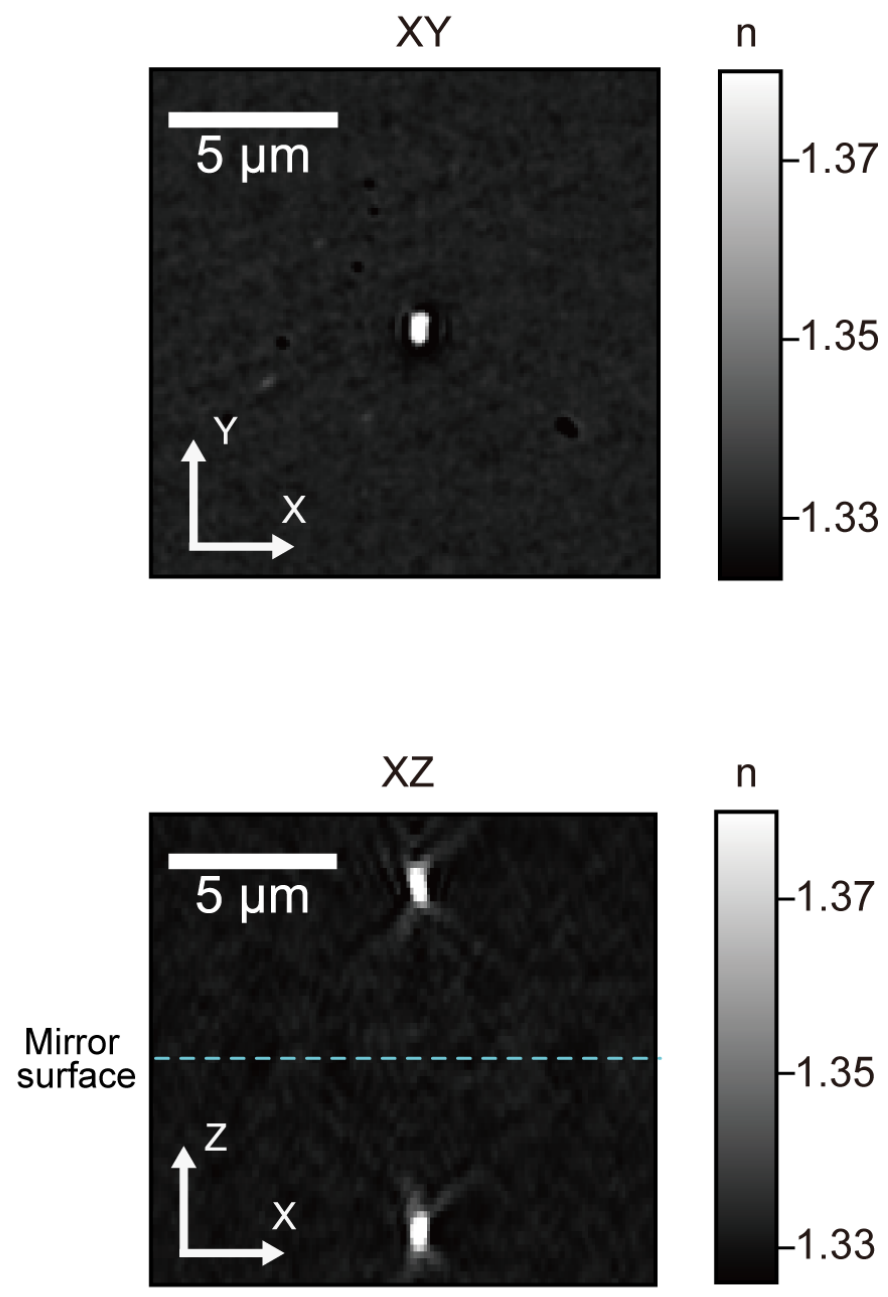

Figure

Reviewer 3's comment #7:

3. System characterization. While the system is characterized, it is not benchmarked against standard or quasi-standard samples. For a newly proposed imaging modality, it is important to establish spatial resolution and contrast performance using well-defined structures. The current demonstrations shows resolution of single lipid droplet, with surrounding media not interfering with the signals (neight scattering nor thermal management, an important parameter for photothermal imaging). As a result, it is difficult to disentangle true resolution limits from sample-dependent effects. Step-function targets or sharp interfaces between two bulks for spatial resolution would be particularly valuable here..

Authors' response:

We thank the reviewer for this important suggestion. We agree that standard or quasi-standard samples are useful for benchmarking the optical reconstruction performance of a new imaging modality. However, the aim of the present study is to establish MIP-ODT for biological chemical imaging. For this purpose, intracellular structures provide a more relevant test sample than isolated standard targets.

This is because the effective spatial resolution in MIP imaging is determined not only by the optical reconstruction performance of ODT, but also by MIR-induced heat diffusion in the local sample environment. Step-function targets or sharp bulk interfaces would be useful for isolating the optical and photothermal response under well-defined conditions, but such samples do not necessarily reproduce the thermal environment of biological cells.

We therefore evaluated the effective MIP resolution using intracellular lipid droplets, which provide spatially localized MIR-absorbing structures embedded in a water-rich cytoplasmic environment. The measured lipid-droplet profiles thus reflect the practical effective resolution under cellular imaging conditions, including both ODT reconstruction performance and photothermal signal broadening.

Reviewer 3's comment #8:

4. Signal metrics. The manuscript emphasizes signal-to-noise ratio as one of a key performance metric. However, for chemically selective imaging, signal-to-background ratio is often more relevant. Demonstrating strong chemical contrast requires clear suppression of background signals, which is not convincingly shown in the current data.

Authors' response:

We thank the reviewer for this comment. We agree that the signal-to-background ratio is an important metric for chemically selective imaging. To address this point, we have added a signal-to-background ratio analysis to Fig. 3. Specifically, we quantified the MIP signal of a cytoplasmic particle relative to the spatially averaged background signal in the off-resonant 3,106 cm^{-1} image, yielding an SBR of 56. This result demonstrates that the resonant MIP signal is clearly distinguishable from the off-resonance background. We have revised the corresponding text accordingly.

Reviewer 3's comment #9:

Minor Comments:

1. Terminology and claims. The term "real-time hyperspectral imaging" is not used accurately. First, the method employs discrete wavelengths and is therefore more appropriately described as spectral rather than hyperspectral imaging. Second, the reported acquisition time (~1 second per spectral stack) does not meet the conventional definition of real-time or video-rate spectral imaging (>20 frames per second).

Authors' response:

We thank the reviewer for this helpful comment. In the original manuscript, we used the term "real-time" to describe the acquisition of a complete spectral stack in approximately 1 s, rather than to imply video-rate acquisition of more than 20 spectral stacks per second. We recognize, however, that this terminology could be interpreted differently. To avoid ambiguity, we have removed "real-time" and now report the acquisition speed explicitly as approximately 1 s per spectral stack, using "high-speed" where appropriate.

Regarding the term "hyperspectral," we note that spectral imaging data are generally acquired at discrete spectral channels. Therefore, the use of discrete excitation wavenumbers alone does not distinguish multi-wavenumber spectral imaging from hyperspectral imaging. Nevertheless, we agree that the spectral dimensionality of the present implementation, which comprises approximately 20 channels across the C–H stretching region with an excitation bandwidth of approximately 10–15 cm^{-1}, may not fully justify the term "hyperspectral." To avoid ambiguity and

adopt more precise terminology, we have replaced “hyperspectral imaging” with “broadband multi-wavenumber spectral imaging” throughout the revised manuscript.

Reviewer 3’s comment #10:

2. Several figures include elements that are not adequately described in the text or captions. For example:

a. Figure 3c: the acquisition time point is not specified.

b. Figure 4a: the meaning of the dotted circle is unclear.

Authors’ response:

We thank the reviewer for pointing this out. We have revised the figure captions to clarify these points. Specifically, we now state the acquisition time points for Fig. 3c and describe the meaning of the dotted circle in Fig. 4a.

Reviewer 3’s comment #11:

3. The discussion of photothermal imaging omits a foundational reference. While many follow-up works are cited, the original demonstration of the technique should be included -https://doi.org/10.1364/OE.19.001378

Authors’ response:

We thank the reviewer for pointing this out. We have added the original demonstration of photothermal imaging as a foundational reference in the revised manuscript.

Reviewer 3’s comment #12:

4.The manuscript does not adequately represent the current state of the field. For example, CARS is not the most recent advancement in this space. More recent developments should be acknowledged, including:

a. Upconversion imaging using external media - https://doi.org/10.1364/JOSAA.393580

b. Non-degenerate two-photon absorption -based imaging, which enables volumetric and spectral modalities using standard semiconductor detectors

- https://doi.org/10.1364/OPTICA.426199 and https://www.science.org/doi/10.1126/sciadv.ade4247

Authors’ response:

We thank the reviewer for this helpful suggestion. We would first like to clarify that the approaches highlighted by the reviewer are conceptually distinct from the present work and are not directly aligned with the primary focus of our Introduction, namely biological chemical imaging with submicrometer spatial resolution. These methods detect MIR light directly or transduce it through nonlinear optical processes, and their spatial resolution therefore remains fundamentally constrained by the diffraction limit at the MIR wavelength. By contrast, MIP-ODT converts local MIR absorption into a photothermally induced refractive-index change and detects this response using visible-light ODT, enabling three-dimensional MIR absorption imaging with spatial resolution governed by the diffraction limit at the visible probe wavelength.

Nevertheless, we agree that these studies represent important recent advances in the broader field of MIR imaging and should be acknowledged. We have therefore added the suggested references, including work on correlative MIR optical coherence tomography and chemical imaging, as well as nondegenerate two-photon absorption approaches for volumetric and spectral MIR imaging.

To Reviewer 1

We are grateful to the Reviewer for taking the time to review our manuscript and give us his/her valuable comments. Our point-by-point responses to the Reviewer's comments are shown below.

Reviewer 1's comment #1:

The authors have satisfactorily answered the reviewers' comments and carefully revised the manuscript. I recommend publication.

Authors' response:

We sincerely thank the Reviewer for the careful evaluation of our revised manuscript and for recommending publication.

To Reviewer 3

We are grateful to the Reviewer for taking the time to review our manuscript and give us his/her valuable comments. Our point-by-point responses to the Reviewer's comments are shown below.

Reviewer 3's comment #1:

The authors have addressed most of my comments, and while a few questions remain, I do not believe they affect the robustness of the study or the validity of the results presented. Overall, I find this to be a solid and well-executed piece of work.

My only remaining concern is whether the manuscript is a sufficiently strong fit for Nature Communications. Similar to Reviewer 2, I remain somewhat concerned about the level of novelty expected for this journal. In my view, the present study represents primarily a logical and relatively routine extension of the authors' previous work rather than a sufficiently substantial conceptual advance.

Authors' response:

We thank the reviewer for their careful assessment and for recognizing the robustness of our study. While building on our previous work, the key challenge was not faster angular scanning alone, but reliably detecting weak photothermal phase changes during rapid illumination switching. This required synchronized single-pulse probing, suppression of SLM-induced phase noise and coherent artifacts, and direct differential tomographic reconstruction to suppress interferometer-induced reconstruction artifacts. These developments enable volumetric chemical imaging without temporal averaging at 19.2 volumes per second, nearly 400 times faster than previous MIP tomography. Crucially, this provides quantitative access to chemically specific, subsecond 3D intracellular dynamics, as demonstrated by lipid-droplet tracking and anomalous-diffusion analysis revealing transport heterogeneity obscured in 2D measurements. We believe that this new measurement capability, rather than the speed increase alone, constitutes the central advance of our work.
